# MTHFR allele and one-carbon metabolic profile predict severity of COVID-19

**DOI:** 10.1073/pnas.2509118122

**Published:** 2025-12-18

**Authors:** Boryana Petrova, Caitlin Syphurs, Andrew J. Culhane, Jing Chen, Ernie Chen, Chris Cotsapas, Denise Esserman, Ruth R. Montgomery, Steven H. Kleinstein, Kinga K. Smolen, Kevin Mendez, IMPACC Network, Jessica Lasky-Su, Hanno Steen, Ofer Levy, Joann Diray-Arce, Naama Kanarek

**Affiliations:** ^a^Department of Pathology, Boston Children’s Hospital, Boston, MA 02115; ^b^Harvard Medical School, Boston, MA 02115; ^c^Core Facilities, Medical University of Vienna, 1090 Vienna, Austria; ^d^Precision Vaccines Program, Boston Children’s Hospital, Boston, MA 02115; ^e^Research Computing, Department of Information Technology, Boston Children’s Hospital, Boston, MA 02115; ^f^Department of Neurology, Yale School of Medicine, New Haven, CT 06510; ^g^Department of Genetics, Yale School of Medicine, New Haven, CT 06510; ^h^Yale School of Public Health, New Haven, CT 06510; ^i^Department of Internal Medicine, Yale School of Medicine, New Haven, CT 06511; ^j^Department of Pathology, Yale School of Medicine, New Haven, CT 06511; ^k^Program in Computational Biology and Biomedical Informatics, Yale University, New Haven, CT 06511; ^l^Channing Institute of Medicine, Brigham and Women’s Hospital, Boston, MA 02115; ^m^Broad Institute of MIT and Harvard, Cambridge, MA 02142

**Keywords:** long COVID, severe COVID risk factors, genetic predisposition, plasma metabolic signature, MTHFR

## Abstract

While the public health burden of SARS-CoV-2 infection has lessened due to natural and vaccine-acquired immunity, emergence of less virulent variants, and antiviral medications, COVID-19 continues to take a significant toll. There are thousands of new hospitalizations and hundreds of deaths per week in the United States, many of whom develop long COVID. Early identification of individuals at high risk of severe COVID-19 is key for monitoring and supporting respiratory status and improving outcomes. Therefore, precision tools for early detection of patients at high risk of severe disease can reduce morbidity and mortality. Here, we report an untargeted, longitudinal plasma metabolomics study of COVID-19 patients. One-carbon metabolism, a pathway previously shown as critical for viral propagation and disease progression, and a potential target for COVID-19 treatment, scored strongly as differentially abundant in patients with severe COVID-19. Targeted metabolite profiling revealed that one arm of the one-carbon metabolism pathway, the methionine cycle, is a major driver of the metabolic profile associated with disease severity. Further, genomic data from the profiled patients revealed a genetic contributor to methionine metabolism and identified the C677T allele of the *MTHFR* gene as a preexisting contributor to disease trajectory—patients that show aberrant one-carbon metabolite levels and that are homozygous for the *MTHFR* C677T, have higher incidence of severe COVID. Our results raise the possibility that *MTHFR* variant status may inform precision COVID-19 treatment strategies.

## Significance

Given long COVID’s health burden, identifying at-risk groups and tailoring prevention strategies is vital to reduce long-term morbidity. Leveraging a large longitudinal U.S. adult cohort, we integrated genomics with targeted and untargeted metabolomics, to identify disruption of one-carbon metabolism as a risk factor for long COVID. Focusing on methionine metabolism, we found that combining MTHFR genetic polymorphism data with plasma methionine cycle biomarkers predicts COVID-19 severity and long COVID risk, offering an accessible molecular axis for potential early clinical risk assessment.

While the impact of SARS-CoV-2 has lessened with the advent of vaccines, antivirals, and less pathogenic variants, COVID-19 continues to take a significant toll across the globe (1, 2). There remains an unmet need to better predict who is at greatest risk of severe or long COVID-19 and to define relevant molecular pathways that may be amenable to novel drugs to reduce the impact of COVID-19.

Long COVID has emerged as a significant public health issue. Long COVID is particularly prevalent among hospitalized COVID-19 patients, with some studies reporting that up to 50% experience persistent physical, cognitive, or mental impairments months after their initial diagnosis (3). This condition poses considerable challenges to quality of life, healthcare systems, and economic productivity (4). Multiple risk factors and immune profiles during the acute phase of infection correlate with delayed clinical and functional recovery after hospital discharge (5, 6), and self-reported long COVID symptoms (7). Current prediction models provide good accuracy but lack precision because they incorporate many environmental and clinical factors and were done at the population level (8). The combination of statistically empowered broad studies (7, 8) with studies that inform specific molecular markers may provide a better clinical tool for long COVID prediction and precision, proactive treatment.

Among the pathways that are relevant to host responses to infection is one-carbon metabolism (9) that encompasses metabolic pathways that involve the transfer of a one-carbon moiety from a donor molecule to an acceptor molecule. One-carbon metabolism enables catabolism and synthesis of key metabolites and cellular building blocks including a few amino acids (serine, glycine, glutamine, and histidine), and nucleotides (10). One-carbon metabolism is critical in supporting rapid cell proliferation and is a target of anticancer therapy (11). Similar to rapidly proliferating cancer cells, virus-infected cells also shift their metabolism to support intense demand for nucleic acids to support viral RNA production (12). Further, it was shown that COVID-19 infected cells rely on the host cell’s one-carbon metabolism and are sensitive to its inhibition by the anti-folate methotrexate (9, 13).

Methylenetetrahydrofolate reductase encoded by the gene *MTHFR* is a key enzyme responsible for the conversion of 5,10-methylene THF to 5-methyl THF. 5-methyl THF is a critical form of folate that feeds the methylation cycle for synthesis of methionine and the methyl donor S-adenosyl methionine (SAM). The *MTHFR* gene has been widely studied due to the common polymorphism C677T, found in ~10% of individuals in North America, that causes conversion of valine to alanine, resulting in a hypomorph with reduced enzymatic activity (14). Carriers of the hypomorph manifest increased plasma homocysteine levels, and association with several pathologies, including oncologic, vascular, and metabolic diseases (15).

The potential link between COVID-19 risk and *MTHFR* polymorphism was hypothesized early in the pandemic (16). This hypothesis is further supported by a geographical correlation between COVID-19 spread and the prevalence of *MTHFR* polymorphism (17), and the correlation between SAM levels and disease severity (18). A few case reports also suggest associations between COVID-19 severity and specific complications such as neuroretinopathy or dermatological manifestations and *MTHFR* polymorphism (19–21). Further, *MTHFR* polymorphisms and methylation status have also been investigated as long COVID-19 risk factors (22, 23), though their utility in patient risk stratification remains uncertain.

We leveraged the NIH/NIAID-supported IMmunoPhenotyping Assessments in a COVID-19 Cohort (IMPACC) (24) to investigate the relationship between COVID-19 progression and one-carbon metabolism pathway intermediates. Targeted metabolomics revealed significant disruptions in the methionine cycle in severe COVID-19 cases. Integrated data from *MTHFR* C677T allele status and the detected levels of intermediates of the one-carbon metabolism pathway revealed potential combined predictor of severe COVID-19. Furthermore, we observed marked one-carbon metabolism perturbations in long COVID patients and identified the combination of homozygous *MTHFR* C677T variant with a methionine-centered metabolite signature as a significant prediction tool for COVID-19 severity and long COVID risk. This work supports future application of precision approaches employing integrated genomics and metabolomics to patient stratification for COVID-19 risk management.

## Results

### Metabolite Profiling of Early-Collected COVID-19 Plasma Samples Reveals Correlation between One-Carbon Metabolism and Disease Severity.

**Fig. 1.**
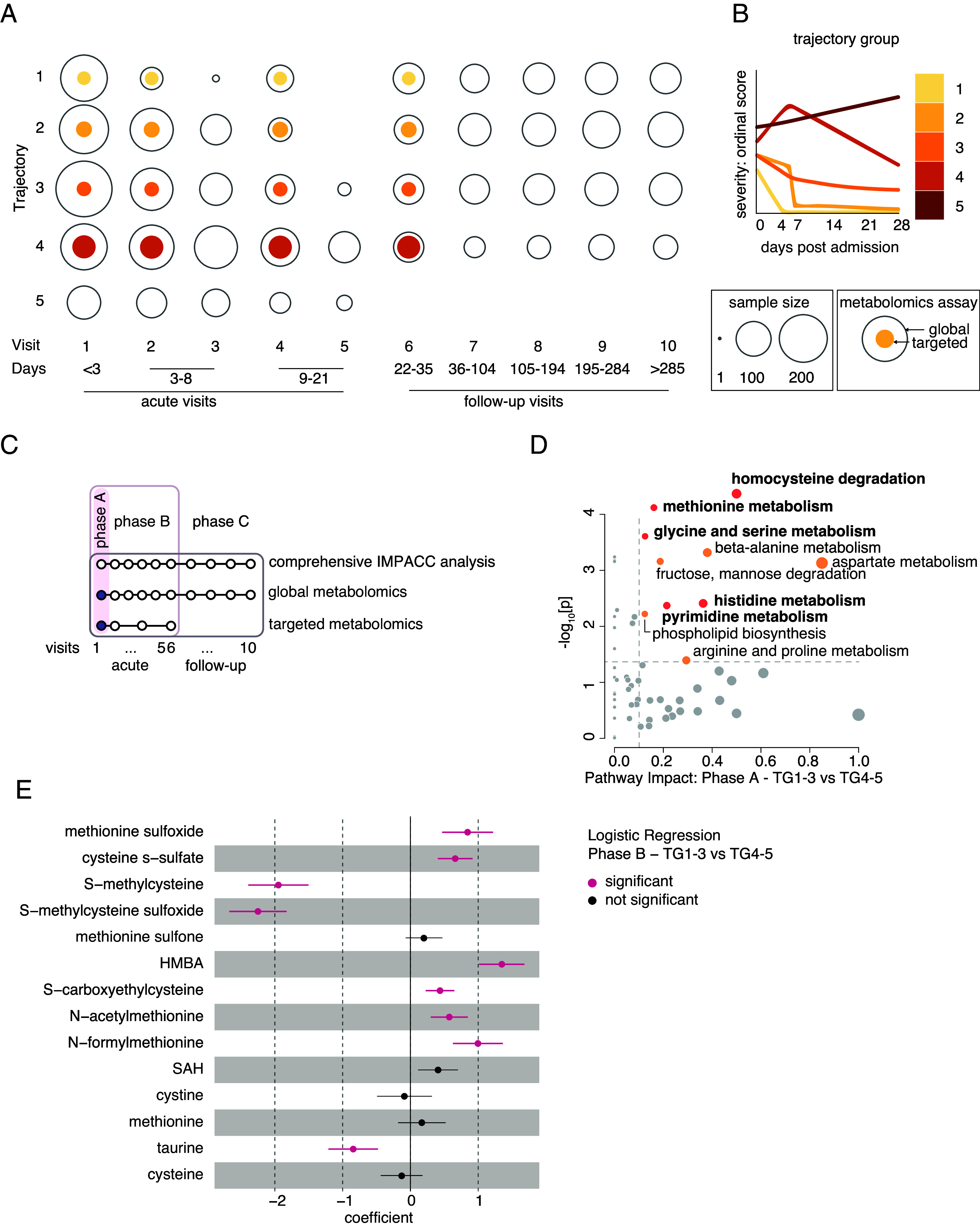
Integrated global and targeted plasma metabolomics of COVID-19 patients reveals one-carbon metabolism association with COVID-19 severity. (*A*) A schematic representing the IMPACC metabolomics cohorts according to trajectory groups and visit number. Days after admission are listed. Filled color-coded area within the circles represents the number of samples analyzed by targeted metabolomics, and open area outlines represent the number of samples analyzed by global metabolomics per visit and per disease trajectory group. (*B*) A schematic depicting the clinical trajectory group (TG) assignments (TG 1-5) for all IMPACC cohort participants (1,164 in total) as reported by Diray-Arce et al. (25). The *x*-axis depicts days since hospital admission, while the *y*-axis displays the ordinal respiratory status score (lowest score indicates mild disease and discharge, highest score indicates mortality). (*C*) A schematic illustrating the study design and employed assays of the IMPACC cohort (24) and outlining the scope of the global and targeted metabolomics assays and the separation into three distinct phases (A-C) encompassing sample collection during hospitalization (“acute”) and during follow-up (“follow up”). Highlighted is Phase A, that provided the data presented in panel *B*, and phase B, that is inclusive of phase A and that provided the data for panel *E*. (*D*) Pathway analysis of differential metabolites from targeted metabolomics profiling of phase A between mild (TG 1-3) and severe (TG 4-5) trajectories performed on the MetaboAnalyst platform, post log-transforming and Pareto scaling mean-centered data. Significantly different pathways (*P* < 0.05) are highlighted as follows: pathways with an impact between 0.1 and 1 are shown with orange circles, and those of particular interest are in dark orange and are highlighted in bold. n = 199. (*E*) Logistic regression analysis from global metabolomics of Phase B for all metabolites within the methionine, cysteine, SAM, and taurine metabolism pathway between mild (TG 1-3) and severe (TG 4-5) trajectory groups. Significantly different metabolites (*P* < 0.01, coefficient > 0) are highlighted in magenta. P-values were adjusted for multiple comparisons using Bonferroni correction (*P* < 0.05). n = 1,055 for participants with metabolomics data.

IMPACC represents a prospective longitudinal study that captured disease progression of 1,035 COVID-19 patients (*SI Appendix*, Table S1) (24). Samples were collected across two phases: acute hospitalization phase and convalescent posthospitalization discharge. Each phase was subdivided into visits: Within the hospitalization stage, there were more frequent visits and longer time intervals between visits within the convalescent phase (Fig. 1*A*). A comprehensive range of clinical, biochemical, immunological, and systems biology assays were performed during both segments as described previously (24). Patients were retrospectively categorized into disease severity trajectories based on the severity of their clinical symptoms with Trajectory Group 1 being the mildest and Trajectory Group 5 being most severe and having a fatal outcome (Fig. 1*B*).

Two different types of metabolomics assays were performed: global and targeted (Fig. 1 *A* and *C* and *SI Appendix*, Fig. S1*A*). Global metabolomics analysis captured all patients at all time points, while targeted metabolomics included a preselected subgroup. The study comprised three phases: phase A analyzed patients’ samples at Visit 1, phase B analyzed samples from Visit 1 through Visit 6 and phase C analyzed all samples within the IMPACC cohort including follow up samples (Fig. 1*C* and *SI Appendix*, Fig. S1*A*). Targeted metabolomics was performed only in phases A and B, whereas phase C was analyzed exclusively by global metabolomics. Both global and targeted metabolomics analyses were used for hypothesis generation and validation. Global metabolomics assays were performed using the platform at Metabolon (25) (Durham, NC), and targeted metabolomic assays were performed at Boston Children’s Hospital (Boston, MA). Additional datasets such as clinical assessments or genomics were accessible through the IMPACC network (5, 24, 26).

To allow for the comparison and integration of data from two different metabolomics platforms, our study relied on metabolomics measurements which partially overlapped between the two platforms, allowing cross reference measurements per patient and visit and determination of a coefficient of correlation (*SI Appendix*, Fig. S1*B*). We compared three abundant metabolites, two of which were common blood indicators (creatine and creatinine) while the third had strong relevance to our study (methionine). All three metabolites showed strong correlation even though liquid chromatography–mass spectrometry (LC–MS) methodology was not synchronized between the two platforms as this was precluded by use of proprietary methods for the global analysis by Metabolon. We performed batch normalization following previously published guidelines from Metabolon (27), applying mean centering across the targeted metabolomics cohort, that successfully eliminated batch effects (*SI Appendix*, Fig. S1*C*).

As samples from Visit 1 became available, and in parallel to continued longitudinal sample collection in following visits, we defined phase A of our study (Fig. 1*C*) and performed targeted metabolomics assay on 199 patient plasma samples collected at Visit 1. We performed pathway analysis of differential metabolites between disease severity trajectories: mild (Trajectory Groups 1-3) and severe (Trajectory Groups 4 and 5) (Fig. 1*D*). This analysis highlighted several pathways involved in one-carbon metabolism and utilization (methionine, histidine, glycine and serine, and homocysteine degradation), that were previously reported to correlate with various aspects of COVID-19 pathology viral replication (9, 12, 13, 28–30). We next analyzed our global metabolomics dataset for data collected at phase B (Fig. 1*C*). We performed logistic regression analysis that demonstrated significantly altered plasma metabolites in the methionine, cysteine, SAM, and taurine metabolism pathways between mild and severe disease trajectory groups (Fig. 1*E*), and across all trajectory groups (*SI Appendix*, Fig. S1*D*). Therefore, our data from two independent metabolomics studies indicate early association between one-carbon metabolism and COVID-19 disease severity and outcome, that remained significant at later time points. This implies that metabolic changes observed as early as Visit 1 are correlated with disease severity and outcome, suggesting potential early markers of disease trajectory or even a predisposition for disease severity rooted in the patient’s metabolic profile.

### Targeted Metabolomics Reveal Methionine Cycle Alterations with COVID-19 Disease Progression and Severity.

**Fig. 2.**
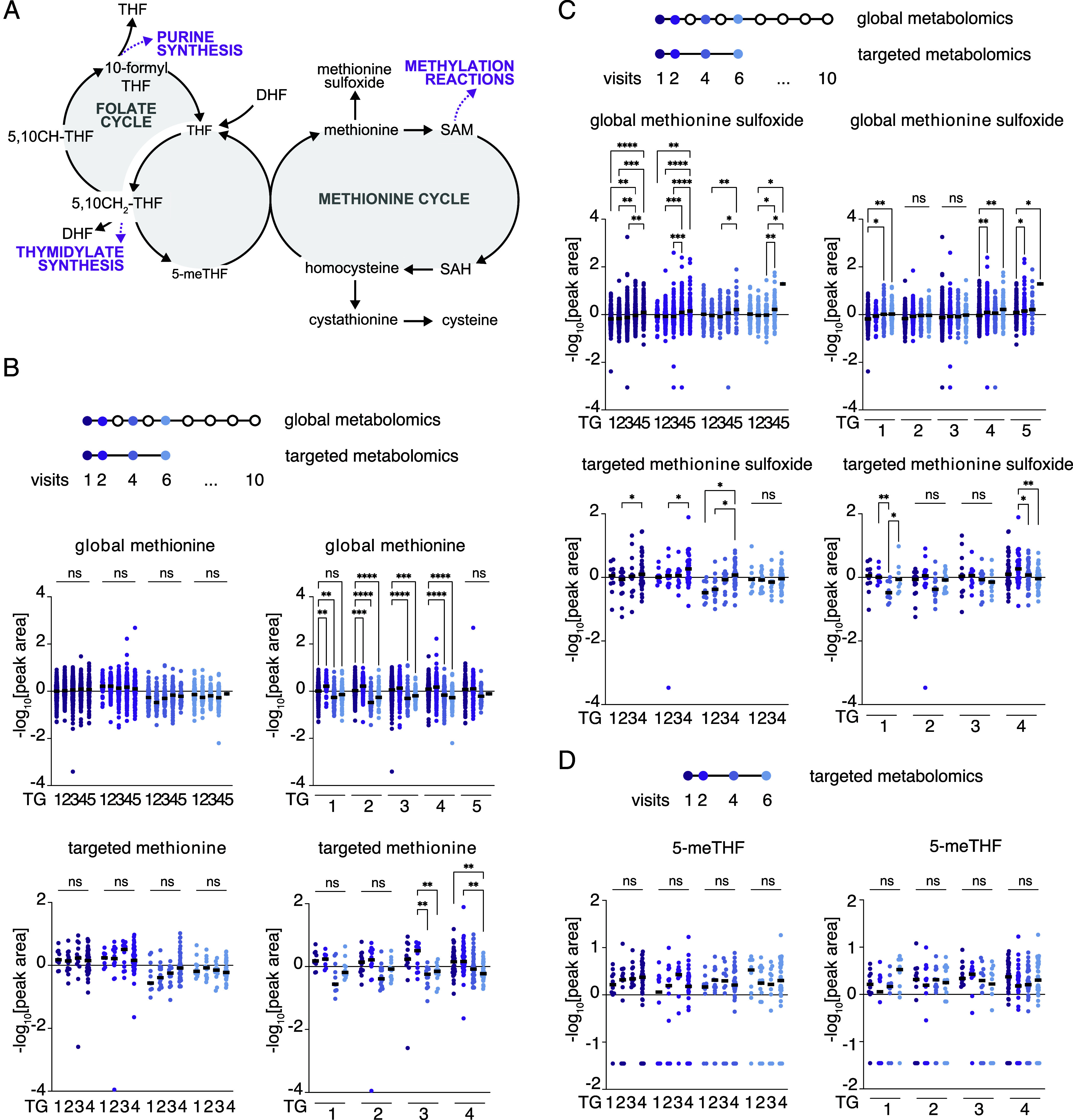
Relative changes in methionine metabolism are correlated with COVID-19 severity. (*A*) Schematic of one-carbon metabolism. Abbreviations are: DHF - dihydrofolate; THF -tetrahydrofolate; 5-meTHF – 5-methyl THF; 5,10-CH THF – 5,10-methenyl THF; 5,10-CH_2_ THF – 5,10-methylene THF; SAM – S-Adenosyl methionine; SAH – S-adenosylhomocysteine. Pathways downstream to the shown reactions are highlighted in magenta. (*B*) Methionine levels from global and targeted metabolomics. At the top a schematic to clarify samples included in the analysis and the visits color code. Below are metabolite profiling data that were mean-centered, log-transformed, and Pareto-scaled. *Left* or *Right* panels are depicting the same data but organized either by trajectory groups (TG1-5) or by visit (V1,2,4 and 6) respectively. A two-way Anova (trajectory comparisons) or a mixed-effects (visit comparisons) analyses were performed, and corresponding *P*-values are indicated where *P < 0.01; **P < 0.001; ***P < 0.001; all remaining comparisons were not significant (ns) and were omitted for clarity. Global metabolomics n = 2,146; Targeted metabolomics n = 199. TG – trajectory group. (*C*) As for *B* but depicting methionine sulfoxide levels. (*D*) 5-methyl THF (5-meTHF) levels from targeted metabolomics. At the top a schematic to clarify samples included in the analysis and the visits color code. Below are metabolite profiling data that were treated and represented as in *B* and *C* except 5-methyl THF was detected only in the targeted analysis. n = 199.

Next, we studied what reactions within one-carbon metabolism-related pathways drive the differential one-carbon metabolism metabolite concentrations and their directionality in severe vs. mild COVID-19 patients. We detected metabolites from both the folate and methionine cycles (Fig. 2*A*); the folate cycle drives nucleotide synthesis with 10-formyl THF as a carbon donor for purine synthesis and 5,10-methylene THF as a carbon donor for thymidylate synthesis (10). The methionine cycle fuels methylation reactions through synthesis of SAM, the methyl group donor of many methyl transferases responsible for methylation of DNA, RNA, and proteins (10, 31). Both global and targeted metabolomics analyses identified alterations in methionine levels in COVID-19 patient plasma (Fig. 2*B*). Specifically, significant changes were observed between visits, while levels did not significantly vary between disease severity trajectory groups. Methionine levels first increased at Visit 2 and then decreased at Visits 4 and 6. This suggested a correlation between methionine levels and COVID-19 disease progression. Further, these data shed light on the inconsistency in reporting methionine levels between studies that involve samples from patients with mild and severe disease, but that were collected at various times following disease onset (12). However, measurements of methionine levels alone could not differentiate between disease severity trajectories at either visit. In contrast, the relative abundance of the methionine oxidation product methionine-sulfoxide was different between plasma samples from patients in different severity trajectory groups as well as throughout consecutive hospital visits especially in the more severe trajectories (TG4 and 5) (Fig. 2*C*). These results were consistent in our targeted metabolomics cohort, despite the lower number of samples included in this subset, indicating that these results are platform independent.

Beyond methionine and its oxidation product, we also analyzed the relative levels of other metabolites of the methionine cycle including glycine, serine, and SAH (Fig. 2*A*). Of note, homocysteine levels were not assessed because their detection requires prior reduction and derivatization, which would have interfered with other metabolites on the platform. In smaller cohorts this is done by splitting each sample and running parallel measurements, but this was not feasible in the large cohort we studied here. We noted significant differential levels in glycine between severity trajectory groups especially at Visits 2 and 4 (*SI Appendix*, Fig. S2*A*). Serine demonstrated more consistently significant differences between severity trajectory groups in all visits, with 1.5 to 1.85-fold decreased levels of serine with higher severity. Its relative levels were lower in more severe patients with, for example, a 1.8-fold decrease observed in TG5 vs. TG1 at Visit 1 (*SI Appendix*, Fig. S2*B*). For SAH, significant differential levels were especially pronounced between trajectory group comparisons, while fewer changes were observed within trajectory per visit (*SI Appendix*, Fig. S2*C*). For glycine and serine, the targeted assay results broadly aligned with global metabolomics findings but were underpowered to capture the biological variability, and SAH was not well-detected by targeted metabolomics for these samples.

Next, we performed targeted folate measurements and detected relative plasma levels of the folate form most abundant in plasma, 5-methyl tetrahydrofolate (5-me THF). Despite there being some subtle changes between visits or trajectories, 5-me THF levels were not significantly different (Fig. 2*D*). This suggests that the folate cycle may be less disrupted compared to the methionine cycle, or that 5-me THF homeostasis is more tightly maintained. However, we cannot rule out the possibility that our targeted assay is underpowered, and this result is influenced by the smaller number of samples available for targeted analysis. Overall, our data suggest that early alterations in the methionine pathway, already observed at Visit 1, could be used to predict COVID-19 disease trajectory and outcome.

### Genetic Susceptibility and Alterations in the Methionine Pathway Correlate with Disease Severity.

We next addressed the possibility of a predisposition that might contribute to the differential levels of methionine cycle metabolites as early as Visit 1 (Fig. 1*D*). A possible predisposition is genetic variance, that in the case of methionine metabolism can be the common variant of the *MTHFR* gene that encodes the MTHFR enzyme. MTHFR converts 5,10-methylene THF to 5-methyl THF, linking the folate and the methionine cycles (*SI Appendix*, Fig. S3*A*). We chose to focus on this enzyme because it has a key role in the methionine cycle, that we identified as possibly perturbed in severe COVID, and because polymorphism in this gene is common enough to allow for detection of differential outcome by variant within our cohort. Common genetic variants exist in the *MTHFR* gene, specifically, C677T (rs1801133) and A1298C (rs1801131) (*SI Appendix*, Fig. S3*B*). The more common heterozygous genotype (C/T, here denoted as GA to reflect the amino acid change) is present in ~30 to 40% of the population worldwide. The homozygous mutant genotype (T/T, here denoted as AA) is present in ~10 to 15% of individuals globally, and results in a substitution of alanine with valine at position 222 in the MTHFR enzyme (*SI Appendix*, Fig. S3*B*). This change is reported to reduce the enzyme’s activity to ~30% of its normal levels with a reported impact on carriers’ physiology (15). The A1298C variant also results in reduced enzyme activity, but to a lesser extent (70%, *SI Appendix*, Fig. S3*B*). Because *MTHFR* polymorphism can result in changes in the methionine cycle flux and levels of related metabolites (32), we set to assess whether this common polymorphism plays a role in the methionine metabolite-related predisposition to severe COVID we identified.

We first verified that the *MTHFR* C677T allele distribution of the IMPACC cohort reflects the distribution found in the general population within the United States (33, 34) (*SI Appendix*, Fig. S3*C* and Table S1). Indeed, the IMPACC cohort had a very similar distribution of the MTHFR variants with 13.3% frequency of the AA allele, compared to the previously reported 14.5% in the general US population. We also found comparable allele frequency between severity trajectory groups (*SI Appendix*, Fig. S3*D*). Of note, in the cohort used for the targeted metabolomics analysis we identified a bias toward the AA allele that appeared at 18.4% frequency in Trajectory Group 4 and not found at all in the mild trajectory groups (*SI Appendix*, Fig. S3*E*). This might stem from the overrepresentation of samples in Trajectory Group 4 in this cohort (Fig. 1*A*).

**Fig. 3.**
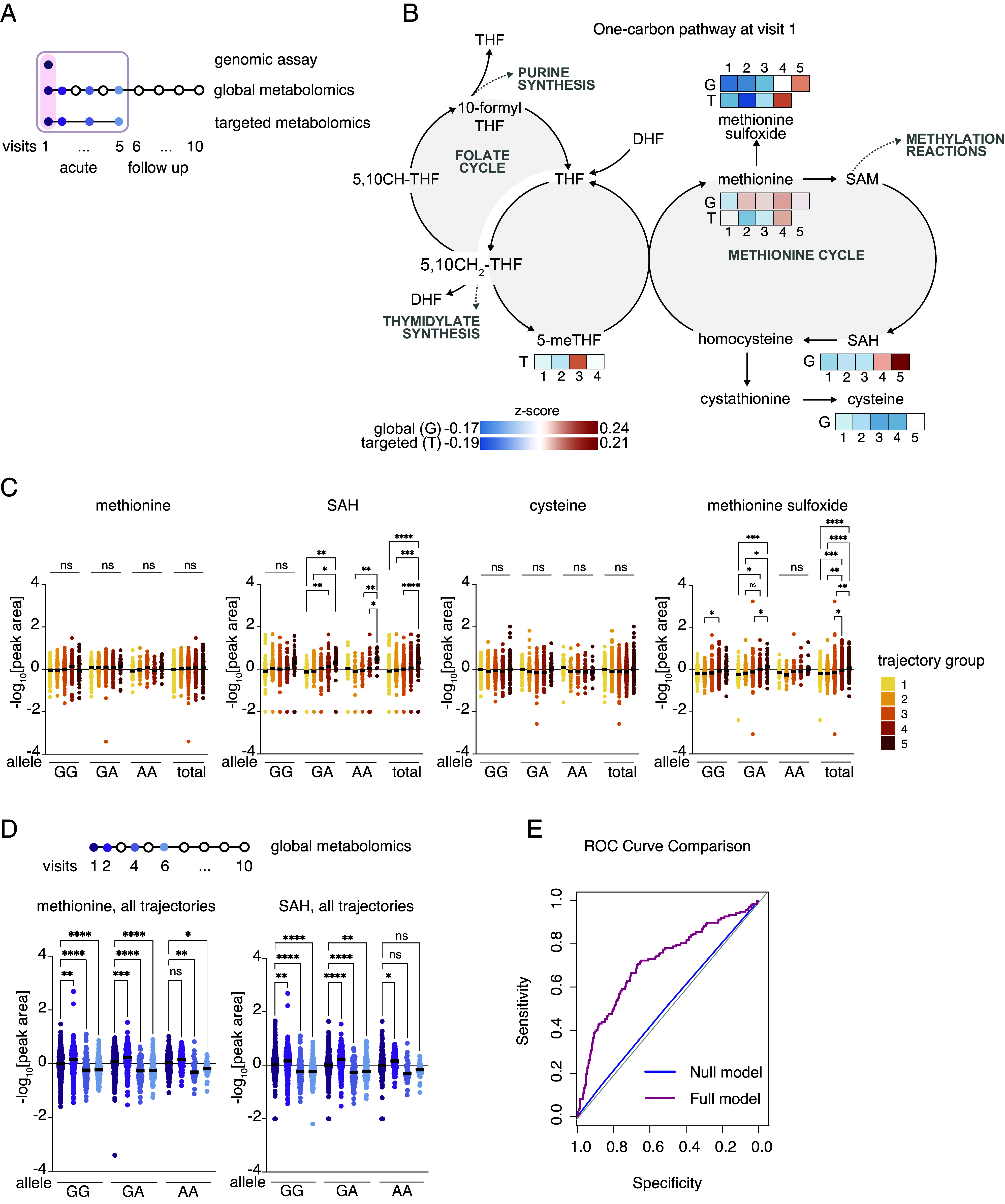
An increase in several metabolites of the methionine pathway at Visit 1 correlates with COVID-19 disease severity and modulation by MTHFR allele status. (*A*) A schematic depicting the visits and analyses of the IMPACC cohort used for data presented in the figure. Analyses include targeted metabolomics, global metabolomics, and genomic assay. (*B*) Schematic of one-carbon metabolism with overlaid differences in abundance between trajectory groups (TG, numbered 1-5) at visit 1 of individual metabolites detected either by global (G) or targeted (T) metabolite profiling. Differences in metabolite abundance between trajectory groups are represented as z-scores, indicated by a color code (see legend). Only metabolites that were detected by either global or targeted metabolomics analysis are represented. (*C*) Methionine, SAH, cysteine, and methionine sulfoxide levels detected by global metabolomics were stratified by MTHFR allele and disease trajectory group. Only data from visit 1 are depicted. Data were mean-centered, log-transformed, and Pareto-scaled. A mixed-effects analysis was performed, and corresponding *P*-values are indicated where *P < 0.01; **P < 0.001; ***P < 0.001; all remaining comparisons were not significant (ns) and were omitted for clarity. GG n = 510; GA n = 358; AA n = 133. (*D*) Methionine and SAH levels detected by global metabolomics stratified by MTHFR allele and visit. Data were mean-centered, log-transformed, and Pareto-scaled. Data from all trajectories were combined. A mixed-effects analysis was performed, and corresponding *P*-values are indicated where *P < 0.01; **P < 0.001; ***P < 0.001; all remaining comparisons were not significant (ns). For methionine and SAH: V1, GG n = 480; V1, GA n = 345; V1, AA n = 129; V2, GG n = 304; V2, GA n = 204; V2, AA n = 82; V4, GG n = 164; V4, GA n = 116; V4, AA n = 36; V6, GG n = 153; V6, GA n = 102; V6, AA n = 31. (*E*) ROC curve goodness of fit comparison likelihood ratio test for null logistic model (predicting mortality based on MTHFR alleles) vs. full logistic model (predicting mortality based on MTHFR alleles and baseline levels of the metabolites SAH, methionine, and methionine-sulfoxide).

Next, we explored whether the C677T polymorphism could influence methionine metabolite status as early as Visit 1. Because individuals with a specific *MTHFR* allele could be assessed as they present to the hospital, we investigated whether *MTHFR* allele information and levels in the methionine cycle metabolites could be used to predict COVID-19 disease outcome and potentially serve as early biomarkers. To this end, we harnessed the IMPACC-wide clinical, genomic, and global metabolomic data (Fig. 3*A*).

Among the methionine cycle metabolites that were detected in samples from Visit 1, methionine sulfoxide and SAH increased significantly with severity while methionine and cysteine were similar between trajectory groups (Fig. 3*B*). When stratified by *MTHFR* allele, for methionine sulfoxide or SAH, there were differences between the individual disease susceptibilities. Namely, for SAH, patients with the AA allele seemed to show similar levels independent of disease trajectory. While for methionine-sulfoxide, significant differences were observed with the GA and AA allele and not the wildtype GG allele (Fig. 3*C*). Changes in the abundance of these metabolites from Visit 1 to later visits were significant in all three genotypes, with lower number of participants likely driving loss of statistical power and reduced significance for the AA group (Fig. 3*D*). Combination of the data for all trajectory groups together resulted in no significant difference in the levels of these metabolites when stratifying our results by *MTHFR* allele status per visit (*SI Appendix*, Fig. S3*F*). When stratifying by trajectory and allele, we observed a reduction in SAH levels in individuals with the AA allele compared to the GG allele at Visits 2 and 4 in Trajectory group 3 (*SI Appendix*, Table S2). These results suggest that *MTHFR* allele status may serve as a predisposition for perturbation of the methionine pathway. However, we were mostly underpowered to use our IMPACC cohort for analysis of *MTHFR* as a sole genetic driver of disease severity and mortality (*SI Appendix*, Fig. S4*A*), or a single parameter for significant hazard ratio, although we saw a trend (*P* = 0.077) toward a higher negative hazard ratio with one of the C677T hypomorphic alleles (*SI Appendix*, Fig. S4*B*). Future studies could assess homocysteine levels—that were not measured in our platform, but that report on both MTHFR allele status and methionine metabolism at the intermediate level—as a potentially accessible clinical marker for COVID-19 severity.

Finally, we assessed whether levels of individual metabolites together with the C677T *MTHFR* allele status at Visit 1 could serve as a prediction factor for mortality risk. We performed likelihood ratio analysis (35–37). This is a statistical method that compares two competing logistic regression models: a null model (baseline, here—*MTHFR* C677T allele status only) and an alternative model (more complex, here—it includes baseline SAH, methionine, and methionine sulfoxide levels in addition to *MTHFR* allele status), and tests whether the additional complexity of the alternative model significantly improves the fit to the data. The inclusion of the three metabolites in the model significantly improved the model’s ability to predict mortality compared to a model based solely on *MTHFR* allele genotype (Fig. 3*E*). The Akaike information criterion (AIC) value decreased from 790.85 (null model) to 738.66 (full model) and the log-likelihood value increased from −392.43 to −363.33, indicating a better fit with the addition of metabolites while accounting for model complexity (χ2(3) = 58.2, *P* < 0.001). In contrast, when performing a separate likelihood ratio analysis, this time adding genetic information to a null model based only on baseline metabolites, the added complexity of the full model did not improve the model’s fit, likely because the genetic contribution is either fully captured by the metabolite data or provides limited additional predictive value in this context (*SI Appendix*, Fig. S4*C*). These findings suggest that the baseline levels of the three metabolites provide additional predictive power beyond *MTHFR* status alone, highlighting their relevance in understanding disease severity.

### A Factor Composed of MTHFR Allele Status and Early Alterations in One-Carbon Metabolism Predicted Long COVID.

**Fig. 4.**
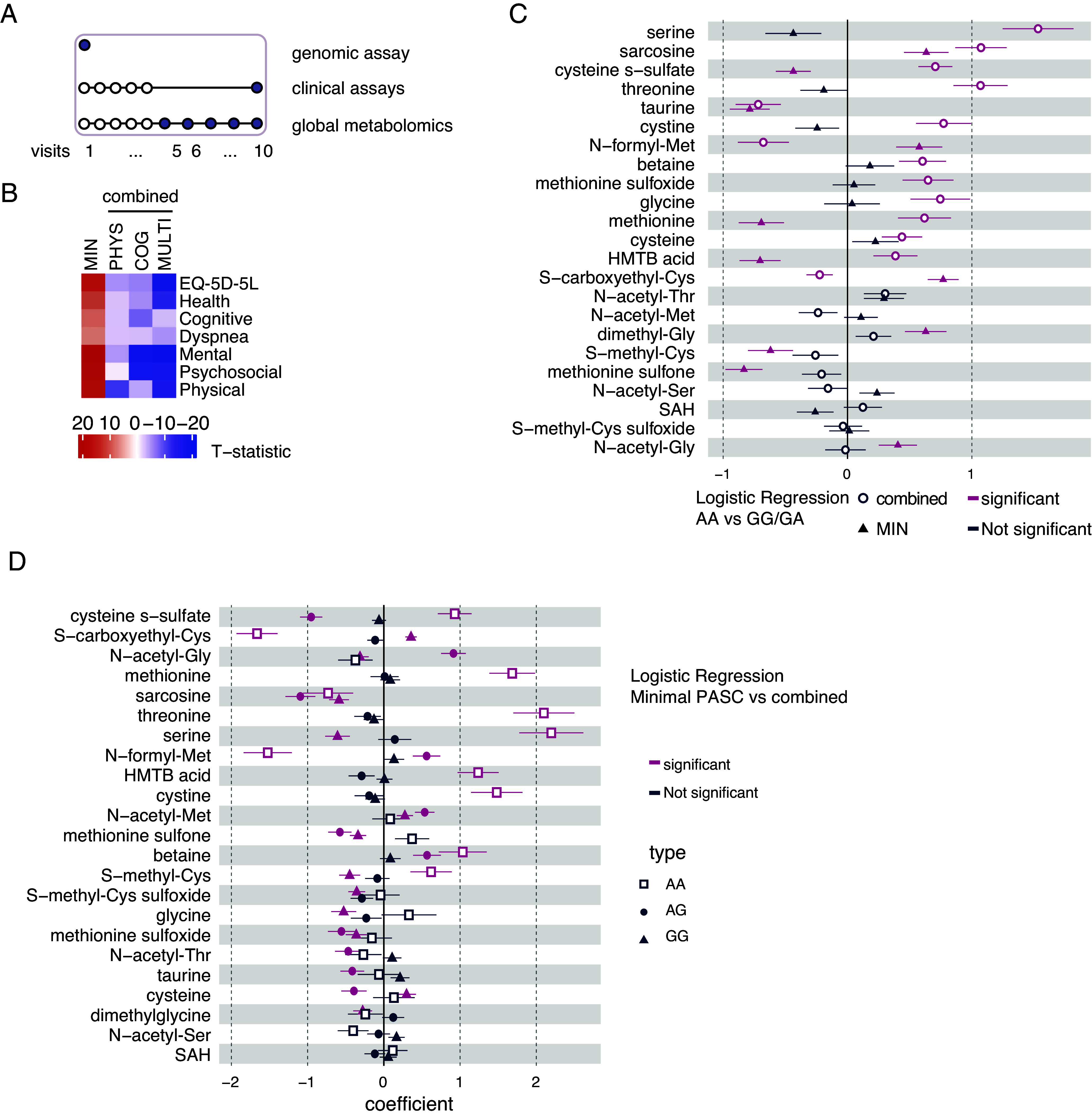
Methionine metabolism alterations during long COVID-19 correlate with MTHFR allele status. (*A*) A schematic illustrating the study design and employed assays of the IMPACC cohort to generate the subsequent analysis in the figure. Data from genomics, global metabolomics, and clinical assays were used. (*B*) A heatmap depicting capture of Long COVID status in the context of the IMPACC study. Relative deficit for each of four convalescent clusters across seven participant-reported outcomes (5) is depicted: EQ-5D-5L, Health Recovery Score (Health), PROMIS Cognitive Function Score (Cognitive), PROMIS Psychosocial Illness Impact Positive Score (Psychosocial), PROMIS Global Mental Health Score (Mental), PROMIS Dyspnea Score (Dyspnea), and PROMIS Physical Function Score (Physical). The color coding denotes a t-statistic comparing the within-cluster mean to the remaining sample mean, with t = 0 denoting the overall sample mean and negative values denoting a deficit. The four clusters were minimal deficit (MIN); physical predominant deficit (PHY); mental/cognitive predominant deficit (COG); and multidomain deficit (MLT). PHY, COG, and MTL were combined into a single measurement of health deficit, “combined,” to generate the subsequent analysis in the figure. PROMIS Patient-Reported Outcomes Measurement Information System. (*C*) Logistic regression analysis for minimal long COVID (triangle), and combined long COVID (open circle) for indicated metabolites from global metabolomics and either wild type and heterozygous MTHFR alleles (GG or GA) or hypomorph alleles (AA), adjusted for sex, age quantile, and BMI. All metabolites within the Creatine Metabolism (n = 3), Glycine, Serine, and Threonine Metabolism (n = 9), and Methionine, Cysteine, SAM, and Taurine Metabolism (n = 14) pathways were included. The analysis included samples from visit 1 of trajectory groups 1 to 4. Significantly different metabolites (*P* < 0.01, coefficient > 0) are in magenta. *P*-values were adjusted for multiple comparisons using Bonferroni correction (*P* < 0.05). AA n = 676; GG/GA n = 3,406. (*D*) Logistic regression analysis for wild type (GG, triangle symbols), heterozygous (GA, circle), or hypomorph (AA, open square) for indicated metabolites from global metabolomics predicting either minimal or combined long COVID clinical outcome, adjusted for sex, age quantile, and BMI. All metabolites within the Creatine Metabolism (n = 3), Glycine, Serine, and Threonine Metabolism (n = 9), and Methionine, Cysteine, SAM, and Taurine Metabolism (n = 14) pathways were included. The analysis included samples from visit 1 of trajectory groups 1 to 4. Significantly different metabolites (*P* < 0.01, coefficient > 0) are in magenta. P-values were adjusted for multiple comparisons using Bonferroni correction (*P* < 0.05). MIN n = 728; combined n = 676.

Next, we tested whether *MTHFR* allele status and early detectable aberrant one-carbon metabolism can be used when combined, for predictions of long COVID, which is a significant public health concern (38, 39). For this purpose, we used patient-reported outcomes (PROs) of IMPACC participants to stratify the cohort by long COVID severity, and we overlaid these data with *MTHFR* allele status and metabolite abundance of one-carbon metabolites detected by our global metabolomics analysis (Fig. 4*A*).

Previously, longitudinal PROs collected during the convalescent period were modeled using latent class mixed models (LCMMs) to identify groups of participants with similar longitudinal patterns (5). PROs were collected using 7 self-reported health survey questionnaires: EQ-5D-5L (European Quality of Life 5 Dimensions 5 Level Version) score (EQ-5D-5L), Health Recovery Score, PROMIS Cognitive Function Score (Cognitive), PROMIS Psychosocial Illness Impact Positive Score (Psychosocial), PROMIS Global Mental Health Score (Mental), PROMIS Dyspnea Score (Dyspnea), and PROMIS Physical Function Score (Physical). More details on the EQ-5D-5L survey can be found in the Methods. To estimate the strength of association of each PRO with each long COVID cluster, t-statistics comparing mean PRO scores of one long COVID cluster across the remaining long COVID clusters were calculated, which identified no associated deficit among three Ward clusters, which were then combined into a singular cluster defined as “minimal.” This resulted in four long COVID clusters which are labeled based on their associations with specific PROs: minimal, physical, mental/cognitive, and multidomain (Fig. 4*B*). The t-statistics were inversely coded so that a negative value indicates a worse self-reported health condition. For patients in the minimal long COVID cluster a large positive t-statistic is observed for all PRO scores—indicating lower mean health deficit scores (where lower score indicates better health) in the minimal long COVID cluster compared to the mean deficits in the other long COVID clusters. The remaining three long COVID clusters were named based on the deficits with negative t-statistics (and therefore greater deficits) for that cluster. In our analysis, we combined the three deficit clusters (physical, mental/cognitive, and multidomain) into a single cluster, referred to as combined from here on (Fig. 4*B*).

We performed logistic regression analysis of metabolite abundance at Visit 1 for metabolites relevant for the one-carbon metabolism pathway comparing the wild type (GG) and heterozygous (GA) alleles of *MTHFR* to the homozygous hypomorph (AA) allele for either the minimal or combined long COVID outcomes (Fig. 4*C*). For patients experiencing long COVID (“combined long COVID”), more metabolites were significantly different if they carried the AA allele. Interestingly, significant changes of some metabolites, such as cysteine s−sulfate, methionine, or HMTB acid, vary in opposite direction in the minimal compared to combined long COVID, suggesting that levels of these metabolites could be adequate biomarkers or to inform on mechanisms of the convalescent disease. This analysis implied that the genetic status of the patient for the *MTHFR* allele is predictive of the metabolic changes observed as early as in Visit 1 for minimal and combined long COVID. Therefore, we asked whether stratification of the IMPACC cohort by *MTHFR* allele status can be predictive of metabolic changes when comparing long COVID outcome (minimal to combined). Indeed, the analysis indicated that patients with the AA allele exhibited greater differences in metabolite levels as early as in Visit 1 when comparing the long COVID outcome of these patients (minimal to combined long COVID) (Fig. 4*D*). The relationship between genotype and metabolite abundance was not uniform across all metabolites with some showing nonlinear patterns across genotypes. This likely reflects the interplay of one-carbon metabolism with other biochemical pathways and the impact of additional genetic and environmental influences. For example, certain metabolites such as cysteine-s-sulfate may reflect oxidative stress-related processes extending beyond MTHFR activity (40, 41). This suggests that patients with the *MTHFR* AA allele manifest different metabolic profiles immediately after hospitalization. We also looked at individual metabolites and noted several significant differences between the metabolite levels in patients with the different alleles depending on long COVID severity (*SI Appendix*, Fig. S5*A*). For example, methionine-sulfone levels at Visit 1 were lower in individuals with the AA allele in the combined long COVID cluster (AA vs. GG W = 1,254, *P* = 0.03; AA vs. AG W = 964, *P* = 0.019) but not in patients who reported minimal deficits. Together our data suggest that *MTHFR* allele status is a genetic predisposition that feeds into early metabolic changes, already observed at Visit 1, and these can predict convalescent COVID-19 outcome. These findings could be valuable for personalized medicine approaches, where understanding the genetic background and metabolic state may inform risk stratification, prognosis, or tailored therapeutic interventions.

## Discussion

COVID-19 continues to place a significant burden on human health and the healthcare system, emphasizing the need to predict which patients may require intensive care, and which are at higher risk for developing long COVID-19. In this study, we identified that intermediates of the methionine cycle are significantly altered during disease progression and vary with COVID severity. By focusing on changes at the time of hospitalization, we demonstrated that the relative concentrations of specific methionine cycle metabolites correlate with disease severity. We found that the common genetic variant *MTHFR* C677T does not fully account for these correlations. Importantly, incorporating *MTHFR* allele status and a predictive model based on significant changes in methionine-related metabolites resulted in an informative predictive model for both disease severity and for the development of long COVID. These findings highlight the potential synergy between genetic and metabolic markers for patient stratification, for informing the need for more aggressive treatment and closer patient observation for patients at risk.

Mechanistically, it is not understood whether perturbed methionine metabolism plays a causal role in the disease trajectory or only reflects it. Our work is not providing an indication for causality, since this is an observational study and we did not introduce a perturbation or another experimental approach that will test the possibility of causality. Therefore, we refrain from suggesting a causal role of perturbed methionine metabolism in COVID-19 pathogenicity. Correlative association between methionine metabolism and COVID-19 severity was reported by another study, that showed that the *MTHFR* gene polymorphism C677T is significantly associated with the severity of COVID-19 (*P* = 0.035, n = 226) (42). Although we cannot prove causality, further research might expose a molecular mechanism that ties the perturbed methionine metabolism to COVID-19 progression and severity. Such a mechanism may be related to the role methionine plays in T cell function (43, 44), critical for effective clearance of SARS-CoV-2 (45). Alternatively, a molecular mechanism that ties methionine metabolism to essential metabolic processes in SARS-CoV-2 propagation is also possible (46). Interestingly, methionine regulates latency for Epstein–Barr virus (EBV) (47), and there is a possibility that EBV activation plays a role in long COVID (48), connecting methionine metabolism to long COVID etiology.

However, in our study, the genetic status of *MTHFR* allele was not sufficient for prediction of severity, and only when genetic and metabolic data were combined, an informative prediction model for disease severity could be structured. We hypothesize that the genetic status of *MTHFR* provides a predisposition, that when combined with other factors results in pathological manifestation that is related (either as a cause or a result) to COVID-19 severity. The factors that can change the manifestation of the metabolic state include environmental, dietary, immunological, infectious disease history, and possibly others (49, 50). These factors contribute to variations in the abundance of key methionine metabolism metabolites, as reflected in our metabolite profiling. This hypothesis is a “two-hit” hypothesis, where *MTHFR* allele status is the first, preexisting, hit, and the metabolite profile at time of hospitalization is the second hit. While the factors that dictate metabolic profile at time of hospitalization are not yet defined, nonetheless, this profile can potentially be used to inform precision care and improve disease outcome.

In this work, we chose to focus on methionine metabolism, but we acknowledge that other pathways are likely to play key roles in COVID severity and allow risk stratification at various stages of disease progression (51–64). We based our choice on the observed significance of the alteration in the methionine pathway (Fig. 1 *D* and *E*), and on the reported biological importance of one-carbon metabolism in COVID-19 propagation (9, 13). Of note, the changes we observed in one-carbon metabolism include several modified metabolites that report on oxidative stress. These include several modifications of cysteine (65) and methionine sulfoxide (66) (Fig. 1*E*). This raises a possibility that the differential levels of these metabolites stem from different redox states in patients at the different disease severity trajectory groups, and not one-carbon metabolism per se. If so, metabolites of the one-carbon metabolism pathway are reporters of oxidative stress and not of the flux through the pathway. Other pathways of interest that are reported here as significantly altered in severe COVID-19 or long COVID can be further studied in the future. Research of interconnection and relationships between pathways is of interest both for mechanistic studies of the viral metabolic needs, and for enhancement and improved accuracy of predictive metabolomic signature for disease severity and long COVID.

Both prolonged hospitalization and greater disease severity have been linked to an increased risk of developing long COVID (6, 42, 67). In a study of long COVID in IMPACC it was found that increased markers of inflammation and heme metabolism are associated with long COVID, and similar to our results, some of these changes can be detected early on and inform follow up and treatment protocols (68). Disruptions in DNA methylation have also been associated with long COVID. Distinct differentially methylated regions (DMRs) have been identified in long COVID patients, which not only distinguish long COVID cases from controls but also stratify long COVID severity, suggesting their potential as biomarkers (22). Our findings fit into this picture and indicate that *MTHFR* genetic variation can influence long COVID symptoms through its impact on methionine cycle metabolites, such as homocysteine. Elevated homocysteine levels, linked to reduced MTHFR activity, contribute to endothelial dysfunction and systemic inflammation (69, 70), which are central to long COVID pathophysiology. These results highlight the intersection of epigenetic changes and metabolic dysregulation in long COVID, underscoring the need for further research to explore their persistence and role in disease progression. This approach may inform strategies to mitigate long-term complications.

IMPACC offers significant advantages, including its large and diverse population drawn from multiple geographically dispersed hospitals across the United States and its longitudinal design, which tracked participants from acute COVID-19 hospitalization through 1 y of postacute recovery. The study’s detailed clinical and biological phenotyping, innovative assays, standardized data analysis pipelines and high-quality data management provided a robust framework for investigating disease mechanisms. Notably, we successfully integrated metabolomics data from two distinct platforms, demonstrating strong agreement between them. However, the study was limited by the absence of assays to explore methylation or other epigenetic effects, which are likely influenced by disruptions in the methylation cycle and one-carbon metabolism. In addition, while we applied both untargeted metabolomics for broad biomarker discovery and targeted metabolomics for focused validation, we understand that neither platform in its current research form is standardized or routinely available in clinical hospital laboratories. The present work provides a foundation for developing a minimal targeted biomarker panel that could be evaluated rigorously for feasibility and cost-effectiveness in clinical settings. Despite our sample size of >1,000 patients, we are still underpowered in genomics data, and other genomics datasets are indeed much bigger. Additional COVID-19 studies, and especially those conducted now, that include longitudinal data on long COVID, can add critical power and can be combined with the data presented here in future studies. Yet, our unique multiomic IMPACC database allowed us to combine the genomics data with metabolomics data for empowerment of our prediction model. Future research can build on our findings to investigate the mechanisms underlying one-carbon metabolism dysregulation and its impact on COVID-19 severity and long COVID phenotypes and address the question of the potential causal role of methionine metabolism dysregulation, as well as the metabolic consequences of this dysregulation on healthy and infected host cells.

Our findings are actionable: Both genetic testing of the gene *MTHFR* and plasma metabolomics can be applied upon hospitalization. As our data suggest that these parameters are informative already at the first hospital visit, early measurement of this risk factor may stratify patients and inform treatment and medical recommendations. Such a personalized approach, combining genetic and metabolite information, can be used to build predictive models for disease severity and long COVID. Prior to clinical implementation, further studies are needed to validate these results and prospectively assess their mechanistic contributions and practical utility.

## Materials and Methods

### Ethics.

NIAID staff conferred with the Department of Health and Human Services Office for Human Research Protections (OHRP) regarding potential applicability of the public health surveillance exception [45CFR46.102 (l) (2)] to the IMPACC study protocol. OHRP concurred that the study satisfied criteria for the public health surveillance exception, and the IMPACC study team sent the study protocol and participant information sheet for review and assessment to institutional review boards (IRBs) at participating institutions. Twelve institutions elected to conduct the study as public health surveillance, while three sites with prior IRB-approved biobanking protocols elected to integrate and conduct IMPACC under their institutional protocols (University of Texas at Austin, IRB 2020-04-0117; University of California San Francisco, IRB 20-30497; Case Western reserve university, IRB STUDY20200573) with informed consent requirements. Participants enrolled under the public health surveillance exclusion were provided information sheets describing the study, samples to be collected, and plans for data deidentification and use. Those that requested not to participate after reviewing the information sheet were not enrolled. Participants did not receive compensation for study participation while hospitalized and subsequently were offered compensation during outpatient follow-up.

### IMPACC Study Cohort.

The IMPACC study enrolled 1,164 unvaccinated patients hospitalized with SARS-CoV-2 infection (confirmed by RT-PCR) from 20 hospitals linked to geographically diverse academic institutions across the United States between May 5th, 2020, and March 19th, 2021 (24, 67). Details regarding the complete study design, clinical and biological sample collection, and participants’ demographics have been previously outlined (37–39). In brief, detailed clinical evaluations and blood samples were gathered within 72 h of hospitalization (visit 1), on days 4, 7, 14, 21, and 28, 90, 180, and 360 following hospital admission (visits 2 to 6, respectively) for the acute phase, and at 3-, 6-, 9-, and 12-mo postdischarge for the convalescent phase (67). NIAID staff conferred with the Department of Health and Human Services Office for Human Research Protections (OHRP) regarding potential applicability of the public health surveillance exception [45CFR46.102 (l) (2)] to the IMPACC study protocol.

#### Host genotyping.

DNA was extracted and samples were genotyped on the Illumina Global Diversity Array as previously described (25). For this analysis, we extracted the MTHFR rs1801133 (C677T) and rs1801131 (A1298AC) polymorphism.

#### Clinical outcome variables.

Group-based trajectory modeling, a likelihood-based approach commonly used to group time series of clinical data and previously described (67), was used to cluster longitudinal measures of the World Health Organization (WHO) seven-point severity ordinal scale into five trajectory groups (TG). Participants with mild disease were defined as those with TG 1-3, and participants with severe disease as those with TG 4-5. TG5 represents all fatal cases within 28-d of admission. Additionally, patients with confirmed mortality at any time during the study were identified.

#### Long COVID patient-reported outcomes assessment.

The EQ-5D-5L survey assesses the participant’s health state through five questions regarding mobility, self-care, usual activities, pain/discomfort, and anxiety/depression, in which participants rate their symptoms for each category on a scale of 1-none to 5-extreme. The Health Recovery Score is a percentage rating from the participant, comparing their health after they were discharged to their health before their COVID-19 infection and hospitalization. The five remaining surveys were from the Common Fund’s Patient-Reported Outcomes Measurement Information System (PROMIS). Using five clustering methods (Ward, McQuitty, Average, PAM, and Complete) and the Gower distance matrix, the Ward algorithm with six clusters was selected as the optimal model based on four fitting statistics (Dunn index, average silhouette width, ratio of within to between sum of squares, and within-cluster sum of squares) (5).

### Global Metabolomics.

Plasma metabolite profiling was conducted by Metabolon using their proprietary standards. Samples were randomized into batches and prepared with Metabolon’s solvent extraction method, adding recovery standards for quality control. Proteins were precipitated with methanol, shaken, and centrifuged. The supernatants were split into five fractions for analysis with UPLC-MS/MS using various ionization modes, including reverse-phase and HILIC. Instruments included a Waters ACQUITY UPLC and a Thermo Scientific Q-Exactive mass spectrometer. Expanded information on randomization and Metabolon data is provided in the *SI Appendix, Extended Methods*.

### Targeted Polar Metabolomics by LC–MS.

Sample preparation for polar and folate detection is described in detail in the *SI Appendix, Extended Methods*. For targeted polar metabolomics, 1 µL reconstituted plasma metabolites (equivalent to 0.05 µL plasma) was injected into a ZIC-pHILIC 150 × 2.1 mm (5 μm particle size) column (EMD Millipore). operated on a Vanquish™ Flex UHPLC Systems (Thermo Fisher Scientific, San Jose, CA, USA). Chromatographic separation was achieved using the following conditions: Buffer A was acetonitrile; buffer B was 20 mM ammonium carbonate, 0.1% ammonium hydroxide in water; resulting pH is around nine without pH adjustment. Gradient conditions we used were 0 to 20 min: linear gradient from 20 to 80% B; 20 to 20.5 min: from 80 to 20% B; 20.5 to 28 min: hold at 20% B at 150 μL/min flow rate. The column oven and autosampler tray were held at 25 °C and 4 °C, respectively. MS data acquisition was performed using a QExactive benchtop orbitrap mass spectrometer equipped with an Ion Max source and a HESI II probe (Thermo Fisher Scientific, San Jose, CA, USA) and was performed in positive and negative ionization mode in a range of m/z = 70 to 1,000, with the resolution set at 70,000, the AGC target at 1 × 10^6^, and the maximum injection time (Max IT) at 20 ms. HESI settings were as follows: Sheath gas flow rate: 35 psi. Aux gas flow rate: 8 psi. Sweep gas flow rate: 1 psi. Spray voltage 3.5 kV (pos); 2.8 kV (neg). Capillary temperature: 320 °C. S-lens RF level: 50 (a.u.). Aux gas heater temperature: 350 °C.

### Data Analysis for Targeted Metabolomics.

Relative quantification of polar metabolites and folate forms was performed with *TraceFinder* 5.1 (Thermo Fisher Scientific, Waltham, MA, USA) using a 7 ppm mass tolerance and referencing an in-house library of chemical standards (220 standards, 7 folate forms, and 37 internal standards). Pooled samples and fractional dilutions were prepared as quality controls and injected at the beginning and end of each run. In addition, pooled samples were interspersed throughout the run to control for technical drift in signal quality as well as to serve to assess the coefficient of variability (CV) for each metabolite. In addition to pooled samples, we interspersed injections of the UPD samples prepared in parallel for each batch to assist in batch normalization and data merging. Data from TraceFinder were further consolidated and normalized manually in Excel. We further followed a normalization strategy (27) to synchronize between Metabolon normalized data and our targeted approach. Briefly, relying on the pooled sample reinjections and dilutions we assigned respectively a CV (coefficient of variation) and an RSQ (r-squared) values to each metabolite per batch. Then performed a filtering step and retained only metabolites for which CV < 30% and RSQ > 0.95 in at least two of the three batches. Finally, for each metabolite values were mean centered across samples and batches. This strategy effectively minimized batch effects and allowed for relative comparisons. Finally, data were log-transformed and Pareto scaled for all multivariate statistics analysis. Individual graphs were plotted in GraphPad (Prism) while pathway analysis, and heatmaps were performed on the MetaboAnalyst 6.0 platform. For the analysis of folate forms similar strategy was followed and post batch normalization folate forms were integrated within polar metabolites dataset. A single outlier was present in batch 1, for which the sample tube was found to contain no plasma. Despite this the tube was processed as a mock sample not to interfere with the strict randomization strategy.

## Supplementary Material

Appendix 01 (PDF)

## Data Availability

Data used in this study is available at ImmPort Shared Data under the accession number SDY1760 (71) and in the NLM’s Database of Genotypes and Phenotypes (dbGaP) under the accession number phs002686.v1.p1 (72). All code is deposited on Bitbucket (https://bitbucket.org/kleinstein/impacc-public-code/src/master/targeted_metabolomics_manuscript/) (73).
