# Supplementary material for: MTHFR allele and one-carbon metabolic profile predict severity of COVID-19
Source: Proc Natl Acad Sci U S A. 2025 Dec 18;122(51):e2509118122. doi: 10.1073/pnas.2509118122 (PMC12745694; doi:10.1073/pnas.2509118122)
Supplement: Supplementary file 1 — Appendix 01 (PDF) [file pnas.2509118122.sapp.pdf]

Supplementary materials for

MTHFR Allele and One-Carbon Metabolic Profile Predict Severity of COVID-19

Boryana Petrova*[1,2,3], Caitlin Syphurs[4], Andrew J Culhane[1], Jing Chen[4,5], Ernie Chen[6], Chris Cotsapas[6], Denise Esserman[7], Ruth R. Montgomery[8], Steven H. Kleinstein[9], Kinga Smolen[4,10], Kevin Mendez[10,11], IMPACC Network[#], Jessica Lasky-Su[10,11], Hanno Steen[1,10], Ofer Levy[4,10,12], Joann Diray-Arce*[4,10], Naama Kanarek*[1,2,12]

1. Department of Pathology, Boston Children's Hospital, Boston, MA 02115, USA
2. Harvard Medical School, Boston, MA 02115, USA
3. Core Facilities, Medical University of Vienna, Austria
4. *Precision Vaccines Program*, Department of Pediatrics, Boston Children's Hospital, Boston, MA, 02115, USA
5. Research Computing, Department of Information Technology, Boston Children's Hospital, Boston, MA 02115, USA
6. Departments of Neurology and Genetics, Yale School of Medicine, New Haven, CT 06510, USA
7. Yale School of Public Health, New Haven, CT 06510, USA
8. Department of Internal Medicine, Yale School of Medicine, New Haven, CT, USA 06511
9. Department of Pathology, Yale School of Medicine, New Haven, CT, USA 06511 and Program in Computational Biology and Biomedical Informatics, Yale University, New Haven, CT, USA 06511
10. Harvard Medical School, Boston, MA 02115, USA
11. Channing Institute of Medicine, Brigham and Women's Hospital, Boston, MA 02115, USA
12. Broad Institute of MIT & Harvard, 415 Main St, Cambridge, MA 02142, USA

* Boryana Petrova, Joann Diray-Arce, Naama Kanarek.

Email: naama.kanarek@childrens.harvard.edu

Author Contributions: Designed research: Boryana Petrova, Joann Diray-Arce, IMPACC Network, Hanno Steen, Ofer Levy, Naama Kanarek; Performed research: Boryana Petrova, Caitlin Syphurs, Andrew J Culhane, Jing Chen; Analyzed data: Boryana Petrova, Caitlin Syphurs, Jing Chen, Kevin Mendez; Contributed tools or reagents: Ernie Chen, Denise Esserman, Joann Diray-Arce, IMPACC Network, Hanno Steen, Chris Cotsapas, Ruth R. Montgomery, Steven H. Kleinstein, Kinga Smolen, Jessica Lasky-Su; Wrote the paper: Boryana Petrova, Caitlin Syphurs, Joann Diray-Arce, Ofer Levy, Naama Kanarek; Provided funding: Joann Diray-Arce, IMPACC Network, Hanno Steen, Ofer Levy, Naama Kanarek

Competing Interest Statement: OL is a named inventor on patents held by Boston Children's Hospital regarding small molecule adjuvants and human *in vitro* systems that model effects of immunomodulators and vaccines. His laboratory is supported in part by sponsored research from *GlaxoSmithKline* (GSK). He is co-founder of and advisor to *ARMR Sciences,* a company that develops approaches to prevent and treat opioid addiction and overdose.

Classification: Biological Sciences: Immunology and Inflammation

Keywords: long COVID; severe COVID risk factors; genetic predisposition; plasma metabolic signature; MTHFR

This PDF file includes:

Expanded Methods
Supplementary Figures S1 to S5

Supplementary Table S1, S2
Group Author information

Expanded Methods

Global Metabolomics additional details

All identified metabolites met Level 1 metabolite identification standards as established by the Chemical Analysis Working Group of the Metabolomics Standards Initiative(1–3). Orthogonal analytical techniques were applied to confirm the identity of key metabolites. Only metabolites with confirmed accurate mass, retention index, and chemical composition were reported.

The raw data consist of metabolites, each annotated to one of nine super pathways: Amino Acid, Carbohydrate, Cofactors and Vitamins, Energy, Nucleotide, Lipid, Peptide, Xenobiotics, and Partially Characterized Molecules. Metabolite levels were measured using LC-MS peak areas, which are proportional to the concentration of each feature.

For quality control, missing values were imputed using half the minimum detected level for each metabolite. Metabolites with zero interquartile range were excluded from further analysis(4). All features underwent log-transformation, normalization, and Pareto scaling to minimize variation in fold-change differences.

Preparation of plasma samples for metabolomics

Plasma samples were collected as described in the IMPACC publication(5) Originally, 500 µL aliquots were flash frozen and stored at -80 ºC in our facility. Aliquots were thawed on ice and re-aliquoted into a new tube at 5 µL to be used for polar metabolomics and folate forms analysis. At this point mock samples were also prepared (empty tubes, left open for the same duration as plasma tubes and handles in parallel) and an aliquot (5 µL) of a universal plasma donor (UPD) mix was thawed. We planned for 3 separate extraction and analysis batches and samples were thawed and aliquoted in subsequent weeks prior to analysis. Per batch, plasma metabolites from a 5 µL aliquot were extracted in 200 µL extraction buffer (80% Methanol, 25 mM Ammonium Acetate and 2.5 mM Na-Ascorbate prepared in LC-MS water, supplemented with isotopically labeled amino acid standards [Cambridge Isotope Laboratories, MSK-A2-1.2], aminopterin, and reduced glutathione standard [Cambridge Isotope Laboratories, CNLM-6245-10]). Samples were vortexed for 10 sec, then centrifuged for 10 minutes at 18,000g to pellet cell debris. The supernatant was transferred into a new tube and dried on ice using a liquid nitrogen dryer. Metabolites were reconstituted in 100 µL water supplemented with QReSS (at 1/1000, isotopically labeled standards [Cambridge Isotope Laboratories, MSK-QRESS-KIT]) and immediately queued for analysis of folate forms. After the folate run was completed (2 days for about 100 samples per batch) samples were queued for analysis of polar metabolites. This ensured that the more unstable folates will be analyzed first.

Detection of folate forms from plasma

For targeted metabolomics of folate forms, 7 µL of reconstituted plasma metabolites (equivalent to 0.62 µL plasma) were injected into an Ascentis Express C18 HPLC column (2.7 um x 15 um x 2.1 mm; Sigma Aldrich). The column oven and autosampler tray were held at 30 °C and 4 °C, respectively. The following conditions were used to achieve chromatographic separation: buffer A was 0.1% formic acid; buffer B was acetonitrile with 0.1% formic acid. The chromatographic gradient was run at a flow rate of 0.250 ml min−1 as follows: 0–5 min: gradient was held at 5% B; 5–10 min: linear gradient of 5% to 36% B; 10.1–14.0 min: linear gradient from 36–95% B; 14.1–18.0 min: gradient was returned to 5% B. The mass spectrometer was operated in full-scan, positive ionization mode using three narrow-range scans: 438–450 m/z; 452–462 m/z; and 470–478 m/z, with the resolution set at 70,000, the AGC target at 10and the maximum injection time of 150 ms. HESI settings were: sheath gas flow rate: 40 psi; Aux gas flow rate: 10 psi; Sweep gas: 0 psi; Spray voltage: 2.8 kV (neg) 3.5 kV (pos); Capillary temperature 300 ºC; S-lens RF level 50 (a.u.); Aux gas heater temperature: 350 ºC.

Supplementary figures

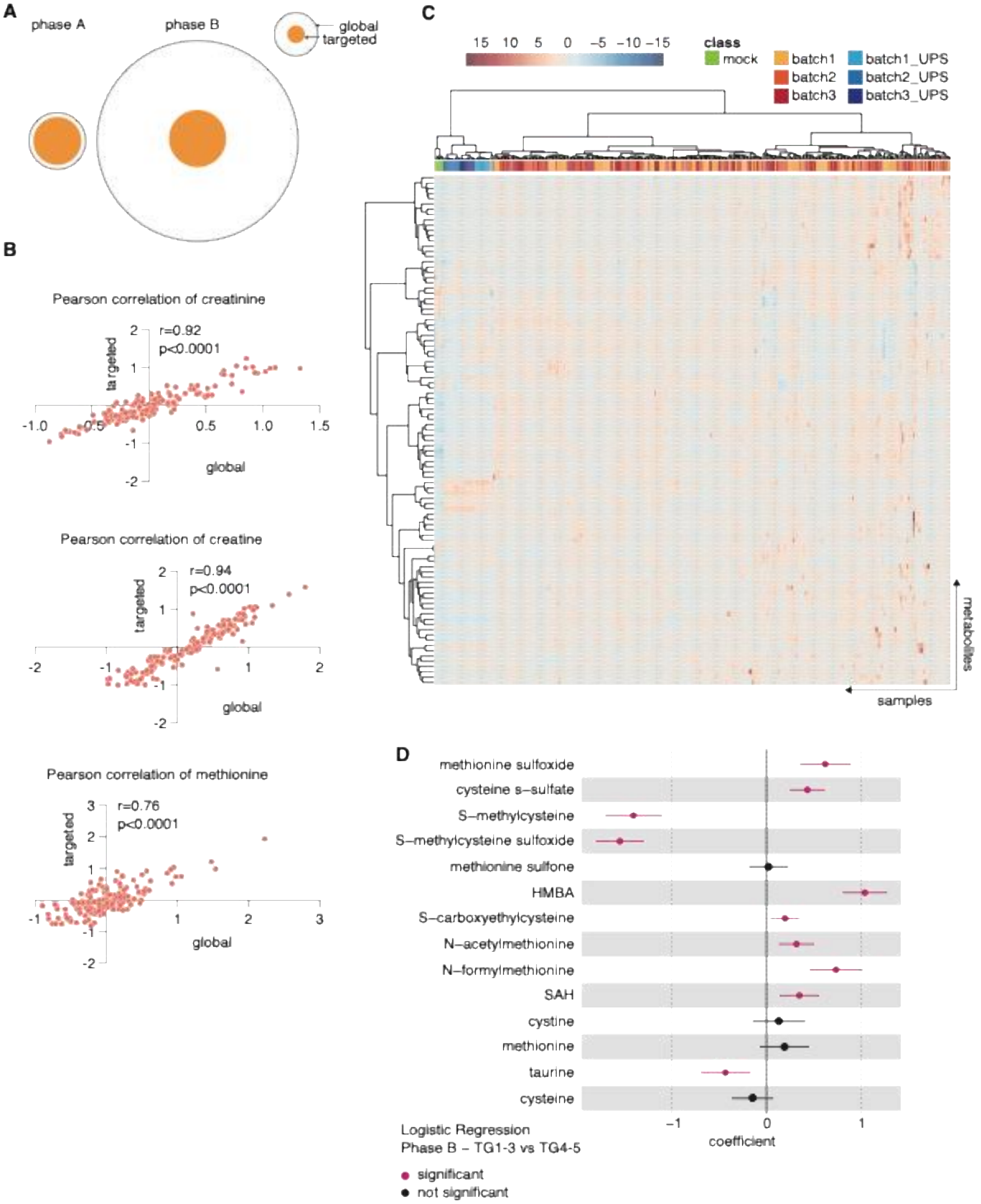
A
phase A
phase B
global
targeted
B
Pearson correlation of creatinine
r=0.92
p<0.0001
targeted
global
Pearson correlation of creatine
r=0.94
p<0.0001
targeted
global
Pearson correlation of methionine
r=0.76
p<0.0001
targeted
global
C
15 10 5 0 −5 −10 −15
class
mock
batch1
batch2
batch3
batch1_UPS
batch2_UPS
batch3_UPS
metabolites
samples
D
methionine sulfoxide
cysteine s−sulfate
S−methylcysteine
S−methylcysteine sulfoxide
methionine sulfone
HMBA
S−carboxyethylcysteine
N−acetylmethionine
N−formylmethionine
SAH
cystine
methionine
taurine
cysteine
Logistic Regression
Phase B − TG1-3 vs TG4-5
coefficient
significant
not significant

Figure S1. Integrated analysis of global and targeted plasma metabolite profiling of the IMPACC cohort.

A A schematic outlining the scope of the global and targeted metabolomics assays as part of phases A and B of the IMPACC study[24]. Circles are depicted at scale to represent the number of samples analyzed in each assay and each phase. Filled color-coded area within the circles represents the number of samples analyzed by targeted metabolomics, and open area outlines represent the number of samples analyzed by global metabolomics. Phase A global n = 1055; Phase B global n = 2146; Phase A targeted n = 199; Phase B targeted n = 303

B Pearson correlation analysis on log-transformed and Pareto-scaled data for the indicated metabolites, comparing the same samples analyzed by both global and targeted metabolomics platforms. The Pearson correlation coefficient (r) and p-values are provided for each metabolite. global n = 2146; targeted n = 303

C Heatmap analysis of differential metabolites between batches 1 to 3 of targeted metabolomics with universal plasma sample (UPS) as associated quality control and mock samples. Analysis was performed on the MetaboAnalyst 6.0 platform, post log-transforming and Pareto scaling mean-centered data.

D Logistic regression (TG1-3 vs TG4-5) analysis from global metabolomics for methionine, cysteine, SAM and taurine metabolism pathway. Significantly different metabolites ($p<0.01$, coefficient >0) are highlighted in magenta. P-values were adjusted for multiple comparisons using Bonferroni correction ($p<0.05$). n=1055

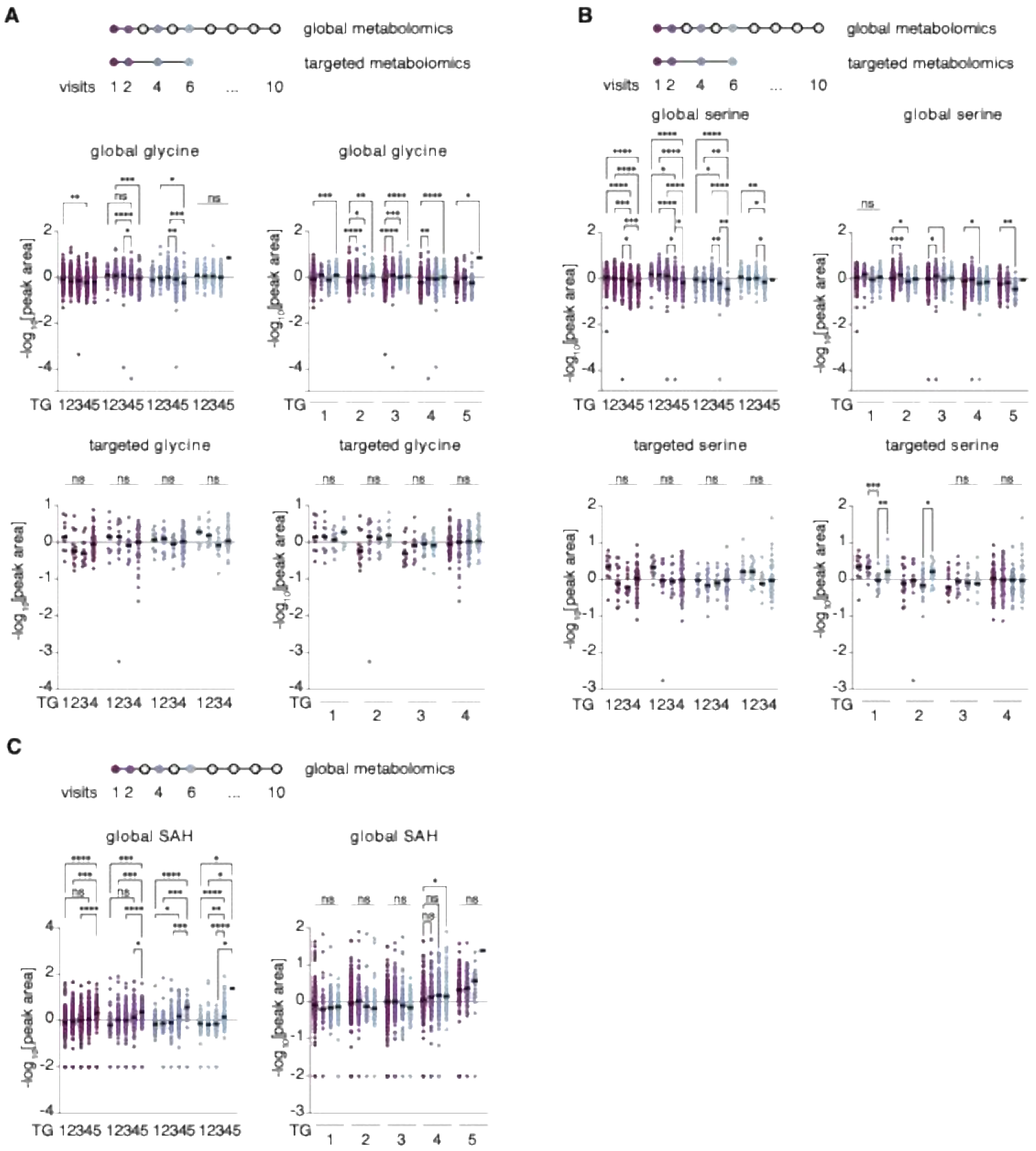

Figure S2. Relative changes of metabolites in the methionine pathway are correlated with COVID-19 severity

A Glycine levels from global and targeted metabolomics. Data were mean-centered, log-transformed, and Pareto-scaled. Left or right panels are depicting the same data organized either by trajectory groups (TG1-5) or by visit (V1,2,4 and 6) respectively. A two-way Anova (trajectory comparisons) or a mixed-effects (visit comparisons) analysis were performed, and corresponding p-values are indicated where *= p<0.01; **= p<0.001; ***= p<0.001; all remaining comparisons were not significant and were omitted for clarity. Global metabolomics n=2146; Targeted metabolomics n=199. TG – trajectory group. V- visit.

B As for A but depicting serine levels.

C S-adenosylhomocystein (SAH) levels from global metabolomics. Data are treated and represented as in A except SAH was detected only in the global metabolomics platform.

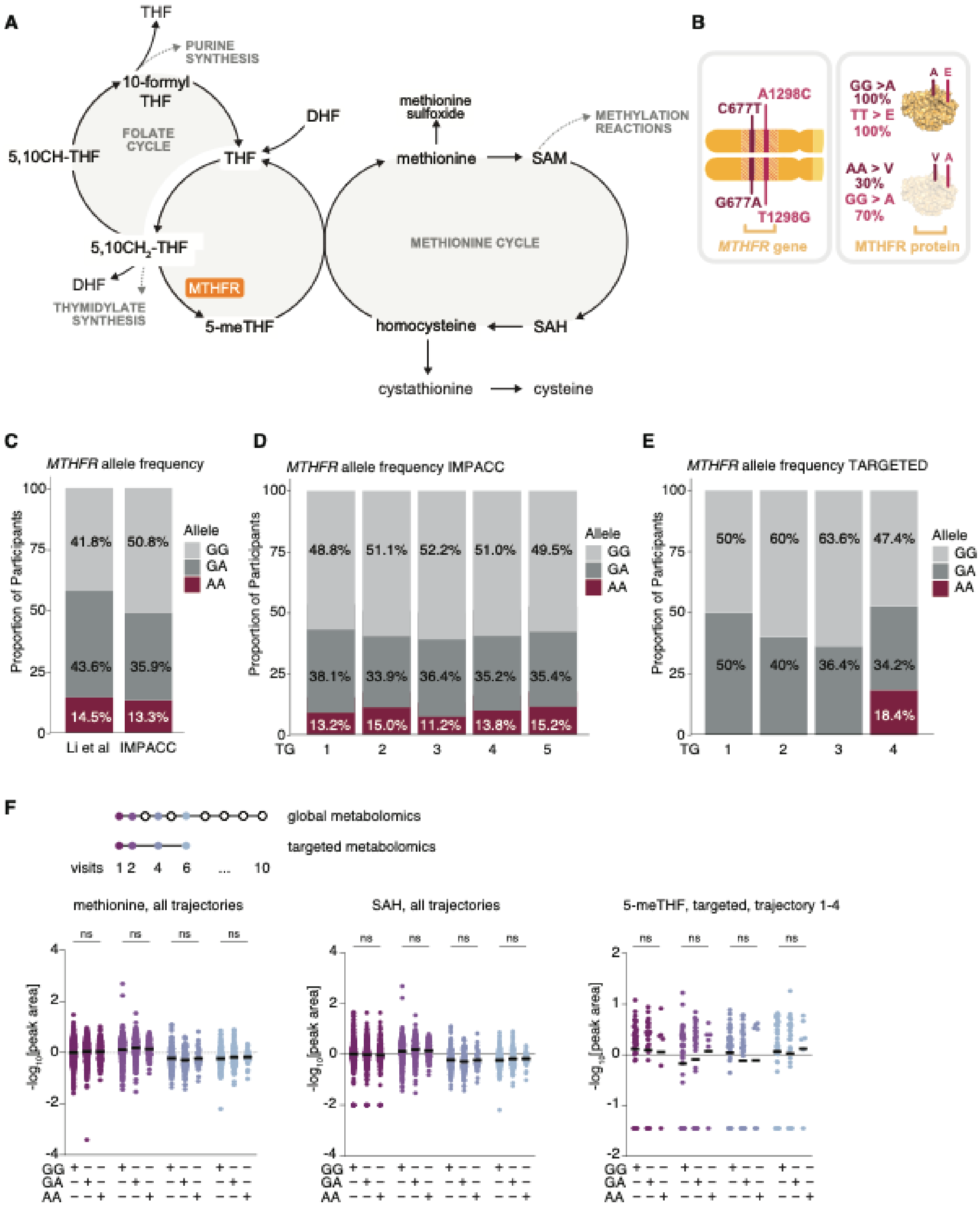
A
THF
PURINE SYNTHESIS
10-formyl THF
FOLATE CYCLE
5,10CH-THF
THF
DHF
5,10CH2-THF
DHF
THYMIDYLATE SYNTHESIS
MTHFR
5-meTHF
methionine sulfoxide
methionine
SAM
METHYLATION REACTIONS
METHIONINE CYCLE
homocysteine
SAH
cystathionine
cysteine
B
C677T
A1298C
G677A
T1298G
MTHFR gene
GG >A 100%
TT > E 100%
AA > V 30%
GG > A 70%
A E
V A
MTHFR protein
C
MTHFR allele frequency
Proportion of Participants
41.8%
50.8%
43.6%
35.9%
14.5%
13.3%
Li et al
IMPACC
Allele
GG
GA
AA
D
MTHFR allele frequency IMPACC
48.8% 51.1% 52.2% 51.0% 49.5%
38.1% 33.9% 36.4% 35.2% 35.4%
13.2% 15.0% 11.2% 13.8% 15.2%
TG 1 2 3 4 5
E
MTHFR allele frequency TARGETED
50% 60% 63.6% 47.4%
50% 40% 36.4% 34.2%
18.4%
TG 1 2 3 4
F
global metabolomics
targeted metabolomics
visits 1 2 4 6 ... 10
methionine, all trajectories
SAH, all trajectories
5-meTHF, targeted, trajectory 1-4
ns
-log10[peak area]
GG
GA
AA

Figure S3. *MTHFR* allele frequency and effects across the IMPACC cohort.

A Schematic of one-carbon metabolism with focus on the enzyme MTHFR. Abbreviations are: DHF - dihydrofolate; THF -tetrahydrofolate; 5-meTHF – 5-methyl THF; 5,10-CH-THF – 5,10-methenyl THF; 5,10-CH_2-THF – 5,10-methylene THF; SAM – S-Adenosyl methionine; SAH – S-adenosylhomocysteine. Downstream one-carbon dependent reactions are indicated.

B Schematic of the hypomorphic *MTHFR* C677T and A1298C alleles (left panel) as well as the residual activity for the corresponding protein mutants (right panel). C677T allele, depicted in dark purple, is at the rs1801133 locus, and is the cause of the A to V amino acid substitution; A1298C allele, depicted in magenta, is at the rs1801131 locus and is the cause of the E to A amino acid substitution.

C Distribution of *MTHFR* C677T allele across the population as reported by Li et al and others[33, 34] in comparison to the IMPACC cohort. GG – wild type allele, GA – C677T heterozygous, AA – C677T homozygous and hypomorph allele.

D, E Distribution of *MTHFR* C677T allele across disease severity trajectory groups in the full IMPACC cohort (D), and in the targeted metabolomics cohort (E). TG – trajectory groups.

F Relative abundance by mass spectrometry of methionine, SAH, and 5-methyl THF. Methionine and SAH were measured using our global metabolomics platform, and 5-methyl THF using our targeted metabolomics. Data was mean-centered, log-transformed, and Pareto-scaled. Data is stratified by *MTHFR* allele and visit, after all trajectories were combined. A two-way Anova was performed, “ns” not significant. For methionine and SAH: V1, GG n = 480; V1, GA n = 345; V1, AA n = 129; V2, GG n = 304; V2, GA n = 204; V2, AA n = 82; V4, GG n = 164; V4, GA n = 116; V4, AA n = 36; V6, GG n = 153; V6, GA n = 102; V6, AA n = 31;
For 5-methyl THF: V1, GG n = 39; V1, GA n = 28; V1, AA n = 6; V2, GG n = 39; V2, GA n = 28; V2, AA n = 6; V4, GG n = 39; V4, GA n = 28; V4, AA n = 6; V6, GG n = 39; V6, GA n = 28; V6, AA n = 6.

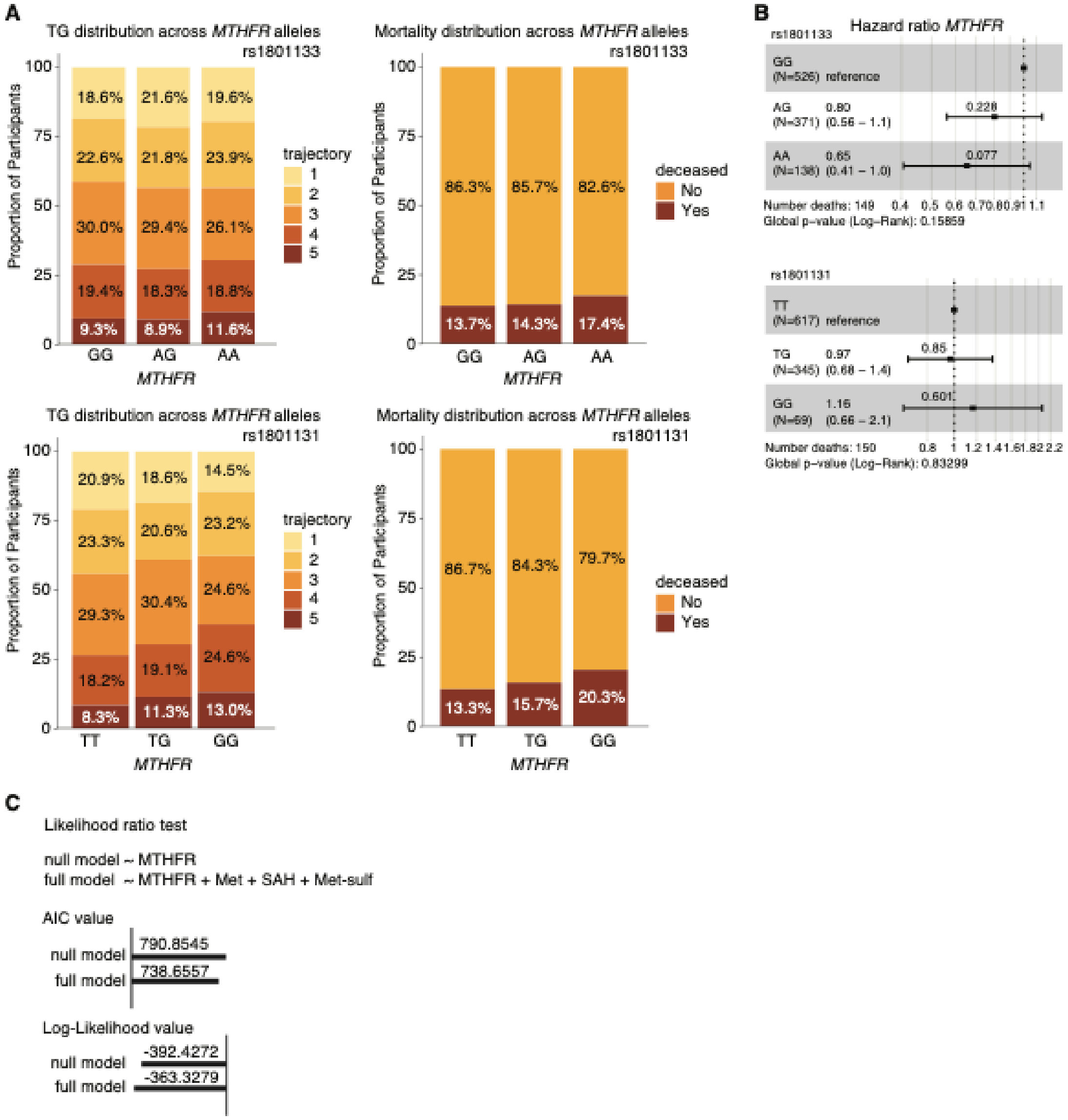

Figure S4. *MTHFR* allele status and correlation with COVID-19 severity

A Frequency of trajectory groups (left panel) or mortality (right panel) across patients with the corresponding *MTHFR* alleles. rs1801133 refers to C677T, rs1801131 refers to A1298C.

B Hazard ratio analysis for *MTHFR* C677T (rs1801133) and A1298C (rs1801131) alleles. Univariate Cox proportional hazards model using the wildtype allele group as a reference. Survival was defined as the time from hospital admission to either death or time of the last follow-up. The p-values of the log-rank test were used to assess significance. For C677T: GG – wild type allele, GA – heterozygous allele, AA – homozygous hypomorph allele. For A1298C: TT – wild type allele, TG – heterozygous allele, GG – homozygous hypomorph allele

C Comparison of AIC and log-likelihood values for the Likelihood ratio test (LRT) results for model comparison in B. The inclusion of the three metabolites SAH, methionine and methionine-sulfoxide, improved model performance compared to the genetic-only (null) model as seen through lower AIC and log-likelihood values for the full model, while adding genetic information to the metabolite-based model did not enhance predictive power.

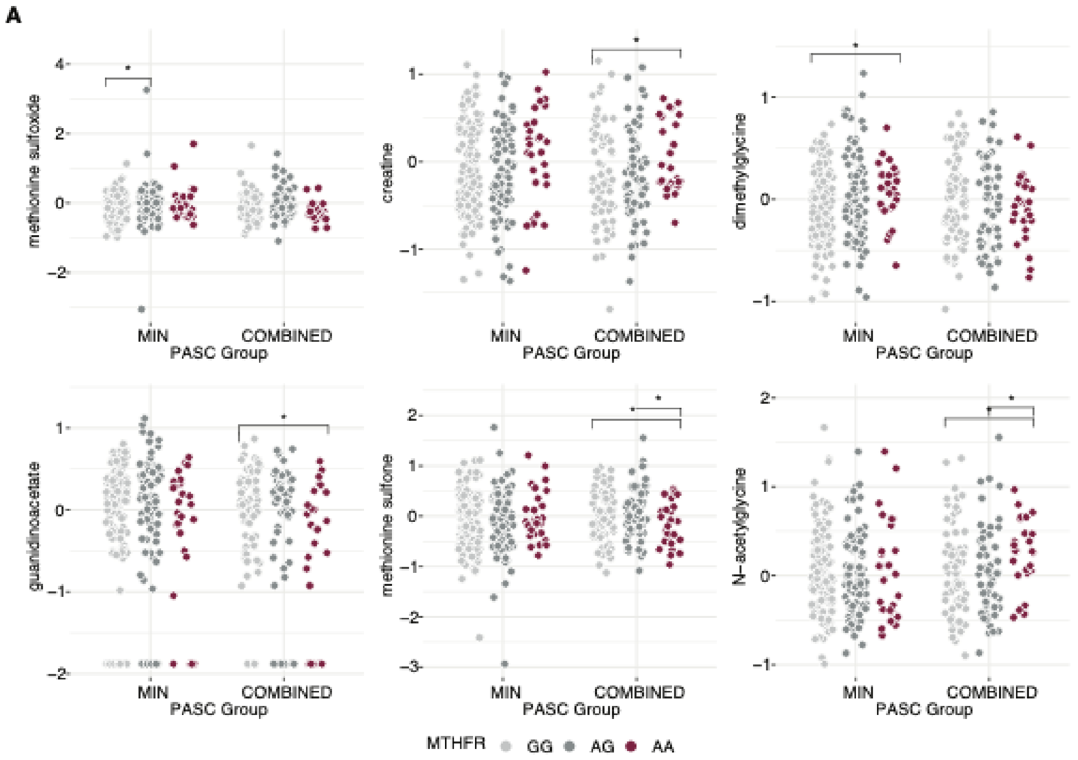

Figure S5. Perturbation of methionine metabolism correlated with long COVID status.
A Levels of the indicated metabolites across *MTHFR* alleles comparing minimal and combined long COVID clinical outcomes. Corresponding Wilcoxon rank sum p-values are indicated where *= p<0.05; all remaining comparisons were not significant and are not depicted.

	Total (N=1035)	GG (N=526)	AG (N=371)	AA (N=138)	P-value
**Sex**					
Male	630 (60.9%)	315 (59.9%)	222 (59.8%)	93 (67.4%)	0.904
Female	405 (39.1%)	211 (40.1%)	149 (40.2%)	45 (32.6%)	
**Age at admission (years)**					
Mean (SD)	58.8 (14.7)	59.3 (14.9)	58.5 (14.8)	57.4 (14.1)	0.668
Median [Min, Max]	60.0 [18.0, 96.0]	61.0 [18.0, 96.0]	59.0 [22.0, 95.0]	57.0 [22.0, 86.0]	
**Visit**					
Visit 1	501 (48.4%)	247 (47.0%)	187 (50.4%)	67 (48.6%)	0.907
Visit 2	204 (19.7%)	96 (18.3%)	69 (18.6%)	39 (28.3%)	
Visit 3	122 (11.8%)	74 (14.1%)	38 (10.2%)	10 (7.2%)	
Visit 4	87 (8.4%)	50 (9.5%)	31 (8.4%)	6 (4.3%)	
Visit 5	21 (2.0%)	9 (1.7%)	8 (2.2%)	4 (2.9%)	
Visit 6	100 (9.7%)	50 (9.5%)	38 (10.2%)	12 (8.7%)	
**Trajectory Group**					
TG1	205 (19.8%)	98 (18.6%)	80 (21.6%)	27 (19.6%)	0.995
TG2	233 (22.5%)	119 (22.6%)	81 (21.8%)	33 (23.9%)	
TG3	303 (29.3%)	158 (30.0%)	109 (29.4%)	36 (26.1%)	
TG4	196 (18.9%)	102 (19.4%)	68 (18.3%)	26 (18.8%)	
TG5	98 (9.5%)	49 (9.3%)	33 (8.9%)	16 (11.6%)	
**Convalescent PASC Group**					
MIN	332 (32.1%)	172 (32.7%)	119 (32.1%)	41 (29.7%)	1
PHY	84 (8.1%)	39 (7.4%)	29 (7.8%)	16 (11.6%)	
COG	69 (6.7%)	35 (6.7%)	24 (6.5%)	10 (7.2%)	
MLT	52 (5.0%)	27 (5.1%)	19 (5.1%)	6 (4.3%)	
Missing	498 (48.1%)	253 (48.1%)	180 (48.5%)	65 (47.1%)	

Table S1. MTHFR allele status of 1,035 COVID-19 patients of the IMPACC cohort, including demographic and disease severity status.

Metabolite	Visit	Trajectory	Comparison	Mean Diff	p-adj	Lower CI	Upper CI	Reject H0	n
methionine	Visit 1	3	AA vs GG	-0.1461	0.0625	-0.2998	0.0077	FALSE	180
methionine	Visit 1	2	AA vs GG	-0.0171	0.8345	-0.1787	0.1445	FALSE	134
methionine	Visit 1	1	AA vs GG	0.027	0.7737	-0.1586	0.2126	FALSE	116
methionine	Visit 1	4	AA vs GG	0.1469	0.1373	-0.0475	0.3413	FALSE	120
methionine	Visit 1	5	AA vs GG	-0.168	0.3264	-0.5077	0.1718	FALSE	59
methionine	Visit 2	3	AA vs GG	-0.1058	0.3193	-0.3156	0.104	FALSE	99
methionine	Visit 2	2	AA vs GG	-0.0524	0.5284	-0.2166	0.1118	FALSE	107
methionine	Visit 2	1	AA vs GG	0.1505	0.5041	-0.3164	0.6175	FALSE	18
methionine	Visit 2	4	AA vs GG	0.041	0.7013	-0.1705	0.2526	FALSE	114
methionine	Visit 2	5	AA vs GG	-0.042	0.8418	-0.4635	0.3794	FALSE	48
methionine	Visit 4	3	AA vs GG	0.0393	0.8261	-0.3206	0.3993	FALSE	40
methionine	Visit 4	2	AA vs GG	-0.4256	0.0554	-0.8619	0.0107	FALSE	25
methionine	Visit 4	1	AA vs GG	-0.2917	0.1949	-0.7398	0.1564	FALSE	37
methionine	Visit 4	4	AA vs GG	0.1953	0.0785	-0.0228	0.4134	FALSE	81
methionine	Visit 4	5	AA vs GG	-0.1179	0.6476	-0.6568	0.4209	FALSE	17
methionine	Visit 6	3	AA vs GG	-0.0335	0.7644	-0.2573	0.1902	FALSE	50
methionine	Visit 6	2	AA vs GG	-0.1438	0.296	-0.4185	0.131	FALSE	39
methionine	Visit 6	1	AA vs GG	-0.1282	0.4849	-0.4945	0.238	FALSE	50
methionine	Visit 6	4	AA vs GG	-0.0415	0.8138	-0.3951	0.312	FALSE	44
SAH	Visit 1	3	AA vs GG	0.0892	0.3831	-0.1121	0.2905	FALSE	180
SAH	Visit 1	2	AA vs GG	0.2132	0.0681	-0.0161	0.4424	FALSE	134
SAH	Visit 1	1	AA vs GG	-0.1203	0.3068	-0.3526	0.1119	FALSE	116
SAH	Visit 1	4	AA vs GG	0.112	0.4008	-0.1511	0.3751	FALSE	120
SAH	Visit 1	5	AA vs GG	-0.2724	0.2293	-0.7211	0.1764	FALSE	59
**SAH**	**Visit 2**	**3**	**AA vs GG**	**0.2958**	**0.0334**	**0.0237**	**0.5678**	**TRUE**	**99**
SAH	Visit 2	2	AA vs GG	0.0053	0.9665	-0.2425	0.253	FALSE	107
SAH	Visit 2	1	AA vs GG	0.3613	0.4574	-0.6445	1.3671	FALSE	18
SAH	Visit 2	4	AA vs GG	-0.0505	0.7223	-0.3314	0.2304	FALSE	114
SAH	Visit 2	5	AA vs GG	-0.0691	0.6047	-0.3358	0.1977	FALSE	48
**SAH**	**Visit 4**	**3**	**AA vs GG**	**0.5146**	**0.0256**	**0.0663**	**0.963**	**TRUE**	**40**
SAH	Visit 4	2	AA vs GG	0.4185	0.1884	-0.2201	1.0572	FALSE	25
SAH	Visit 4	1	AA vs GG	0.2221	0.3351	-0.2392	0.6833	FALSE	37
SAH	Visit 4	4	AA vs GG	0.0618	0.6944	-0.2503	0.374	FALSE	81
SAH	Visit 4	5	AA vs GG	0.0436	0.866	-0.4971	0.5842	FALSE	17
SAH	Visit 6	3	AA vs GG	0.3393	0.082	-0.0448	0.7234	FALSE	50
SAH	Visit 6	2	AA vs GG	0.2005	0.4211	-0.2989	0.6999	FALSE	39
SAH	Visit 6	1	AA vs GG	0.0424	0.8218	-0.3343	0.4191	FALSE	50
SAH	Visit 6	4	AA vs GG	-0.0076	0.9731	-0.4593	0.4441	FALSE	44
cysteine	Visit 1	3	AA vs GG	-0.0239	0.7641	-0.1807	0.1329	FALSE	180
cysteine	Visit 1	2	AA vs GG	0.0457	0.6109	-0.1317	0.2231	FALSE	134
cysteine	Visit 1	1	AA vs GG	-0.1173	0.2224	-0.3067	0.0721	FALSE	116
cysteine	Visit 1	4	AA vs GG	0.0144	0.8927	-0.1967	0.2255	FALSE	120
cysteine	Visit 1	5	AA vs GG	0.0605	0.7772	-0.3656	0.4865	FALSE	59
cysteine	Visit 2	3	AA vs GG	-0.0932	0.4036	-0.3139	0.1274	FALSE	99
cysteine	Visit 2	2	AA vs GG	-0.0039	0.9698	-0.209	0.2011	FALSE	107
cysteine	Visit 2	1	AA vs GG	0.2764	0.3587	-0.3437	0.8966	FALSE	18
cysteine	Visit 2	4	AA vs GG	-0.1083	0.4846	-0.4142	0.1977	FALSE	114
cysteine	Visit 2	5	AA vs GG	0.2083	0.208	-0.12	0.5365	FALSE	48
cysteine	Visit 4	3	AA vs GG	-0.0767	0.638	-0.4042	0.2508	FALSE	40
cysteine	Visit 4	2	AA vs GG	-0.1143	0.6172	-0.581	0.3524	FALSE	25
cysteine	Visit 4	1	AA vs GG	-0.2306	0.2209	-0.6062	0.145	FALSE	37
cysteine	Visit 4	4	AA vs GG	-0.1815	0.3562	-0.5708	0.2078	FALSE	81
**cysteine**	**Visit 4**	**5**	**AA vs GG**	**0.863**	**0.0269**	**0.1132**	**1.6127**	**TRUE**	**17**
cysteine	Visit 6	3	AA vs GG	-0.0069	0.9556	-0.2551	0.2413	FALSE	50
cysteine	Visit 6	2	AA vs GG	-0.0539	0.7012	-0.3366	0.2287	FALSE	39
cysteine	Visit 6	1	AA vs GG	0.1601	0.3013	-0.1479	0.468	FALSE	50
cysteine	Visit 6	4	AA vs GG	0.1652	0.4392	-0.2617	0.5921	FALSE	44
Methionine_Sulfoxide	Visit 1	3	AA vs GG	-0.1105	0.146	-0.2599	0.0389	FALSE	180
Methionine_Sulfoxide	Visit 1	2	AA vs GG	0.049	0.4934	-0.0922	0.1902	FALSE	134
Methionine_Sulfoxide	Visit 1	1	AA vs GG	-0.067	0.384	-0.2189	0.0849	FALSE	116
Methionine_Sulfoxide	Visit 1	4	AA vs GG	-0.0143	0.8862	-0.2123	0.1836	FALSE	120
Methionine_Sulfoxide	Visit 1	5	AA vs GG	-0.1305	0.3659	-0.4173	0.1563	FALSE	59
Methionine_Sulfoxide	Visit 2	3	AA vs GG	-0.0631	0.557	-0.2755	0.1493	FALSE	99
Methionine_Sulfoxide	Visit 2	2	AA vs GG	-0.1788	0.0575	-0.3635	0.0058	FALSE	107
Methionine_Sulfoxide	Visit 2	1	AA vs GG	0.2321	0.3652	-0.2959	0.7602	FALSE	18
Methionine_Sulfoxide	Visit 2	4	AA vs GG	-0.237	0.0769	-0.5	0.026	FALSE	114
Methionine_Sulfoxide	Visit 2	5	AA vs GG	0.1577	0.4672	-0.2753	0.5908	FALSE	48
Methionine_Sulfoxide	Visit 4	3	AA vs GG	-0.054	0.7335	-0.3725	0.2645	FALSE	40
Methionine_Sulfoxide	Visit 4	2	AA vs GG	0.2304	0.2918	-0.2114	0.6723	FALSE	25
Methionine_Sulfoxide	Visit 4	1	AA vs GG	-0.3671	0.1803	-0.9122	0.1781	FALSE	37
Methionine_Sulfoxide	Visit 4	4	AA vs GG	-0.0583	0.7045	-0.3633	0.2466	FALSE	81
Methionine_Sulfoxide	Visit 4	5	AA vs GG	0.405	0.3279	-0.4486	1.2587	FALSE	17
Methionine_Sulfoxide	Visit 6	3	AA vs GG	-0.0463	0.8061	-0.4233	0.3307	FALSE	50
Methionine_Sulfoxide	Visit 6	2	AA vs GG	-0.1597	0.2753	-0.452	0.1326	FALSE	39
Methionine_Sulfoxide	Visit 6	1	AA vs GG	-0.0454	0.8283	-0.4645	0.3736	FALSE	50
Methionine_Sulfoxide	Visit 6	4	AA vs GG	-0.3996	0.055	-0.8081	0.0089	FALSE	44

Table S2. Tukey's multiple comparisons of MTHFR genotypes (GG vs. GA, GG vs. AA) across metabolite concentrations in visits 1, 2, 4, and 6 of the IMPACC cohort, stratified by disease trajectory. Reported values include mean differences, adjusted p-values, confidence intervals, and significance flags.

Group Author Information

#The IMPACC Network

National Institute of Allergy and Infectious Diseases, National Institute of Health, Bethesda, MD 20814, USA:
Patrice M. Becker, Alison D. Augustine, Steven M. Holland, Lindsey B. Rosen, Serena Lee, Tatyana Vaysman

Clinical and Data Coordinating Center (CDCC), Precision Vaccines Program, Boston Children's Hospital, Harvard Medical School, Boston, MA 02115, USA:
Al Ozonoff, Joann Diray-Arce, Jing Chen, Alvin T. Kho, Carly E. Milliren, Annmarie Hoch, Ana C. Chang, Kerry McEnaney, Caitlin Syphurs, Brenda Barton, Claudia Lentucci, Maimouna D. Murphy, Mehmet Saluvan, Tanzia Shaheen, Shanshan Liu, Marisa Albert, Arash Nemati Hayati, Robert Bryant, James Abraham, Mitchell Cooney, Meagan Karoly

Benaroya Research Institute, University of Washington, Seattle, WA 98101, USA:
Matthew C. Altman, Naresh Doni Jayavelu, Scott Presnell, Bernard Kohr, Tomasz Jancsyk, Azlann Arnett

La Jolla Institute for Immunology, La Jolla, CA 92037, USA:
Bjoern Peters, James A. Overton, Randi Vita, Kerstin Westendorf

Knocean Inc. Toronto, ON M6P 2T3, Canada:
James A. Overton

Precision Vaccines Program, Boston Children's Hospital, Harvard Medical School, Boston, MA 02115, USA:
Ofer Levy, Hanno Steen, Patrick van Zalm, Benoit Fatou, Kinga K. Smolen, Arthur Viode, Simon van Haren, Meenakshi Jha, David Stevenson, Athena N. Nguyen, Alec L. Plotkin, Sanya Thomas, Boryana Petrova, Naama Kanarek

Brigham and Women's Hospital, Harvard Medical School, Boston, MA 02115, USA:
Lindsey R. Baden, Kevin Mendez, Jessica Lasky-Su, Alexandra Tong, Rebecca Rooks, Michael Desjardins, Amy C. Sherman, Stephen R. Walsh, Xhoi Mitre, Jessica Cauley, Xiaofang Li, Bethany Evans, Christina Montesano, Jose Humberto Licona, Jonathan Krauss, Nicholas C. Issa, Jun Bai Park Chang, Natalie Izaguirre

Metabolon Inc, Morrisville, NC 27560, USA:
Scott R. Hutton, Greg Michelotti, Kari Wong

Prevention of Organ Failure (PROOF) Centre of Excellence, University of British Columbia, Vancouver, BC V6T 1Z3, Canada:
Scott J. Tebbutt, Casey P. Shannon

Case Western Reserve University and University Hospitals of Cleveland, Cleveland, OH 44106, USA:
Rafick-Pierre Sekaly, Slim Fourati, Grace A. McComsey, Paul Harris, Scott Sieg, George Yendewa, Mary Consolo, Heather Tribout, Susan Pereira Ribeiro

Drexel University, Tower Health Hospital, Philadelphia, PA 19104, USA:
Charles B. Cairns, Elias K. Haddad, Michele A. Kutzler, Mariana Bernui, Gina Cusimano, Jennifer Connors, Kyra Woloszczuk, David Joyner, Carolyn Edwards, Edward Lee, Edward Lin, Nataliya Melnyk, Debra L. Powell, James N. Kim, I. Michael Goonewardene, Brent Simmons, Cecilia M. Smith, Mark Martens, Brett Croen, Nicholas C. Semenza, Mathew R. Bell, Sara Furukawa, Renee McLin, George P. Tegos, Brandon Rogowski, Nathan Mege, Kristen Ulring, Pam Schearer, Judie Sheidy, Crystal Nagle

MyOwnMed Inc., Bethesda, MD 20817, USA:
Vicki Seyfert-Margolis

Emory School of Medicine, Atlanta, GA 30322, USA:
Nadine Rouphael, Steven E. Bosinger, Arun K. Boddapati, Greg K. Tharp, Kathryn L. Pellegrini, Brandi Johnson, Bernadine Panganiban, Christopher Huerta, Evan J. Anderson, Hady Samaha, Jonathan E. Sevransky, Laurel Bristow, Elizabeth Beagle, David Cowan, Sydney Hamilton, Thomas Hodder, Amer Bechnak, Andrew Cheng, Aneesh Mehta, Caroline R. Ciric, Christine Spainhour, Erin Carter, Erin M. Scherer, Jacob Usher, Kieffer Hellmeister, Laila Hussaini, Lauren Hewitt, Nina Mcnair, Susan Pereira Ribeiro, Sonia Wimalasena

Icahn School of Medicine at Mount Sinai, New York, NY 10029, USA:
Ana Fernandez-Sesma, Viviana Simon, Florian Krammer, Harm Van Bakel, Seunghee Kim-Schulze, Ana Silvia Gonzalez-Reiche, Jingjing Qi, Brian Lee, Juan Manuel Carreño, Gagandeep Singh, Ariel Raskin, Johnstone Tcheou, Zain Khalil, Adriana van de Guchte, Keith Farrugia, Zenab Khan, Geoffrey Kelly, Komal Srivastava, Lily Q. Eaker, Maria C. Bermúdez-González, Lubbertus C.F. Mulder, Katherine F. Beach, Miti Saksena, Deena Altman, Erna Kojic, Levy A. Sominsky, Arman Azad, Dominika Bielak, Hisaaki Kawabata, Temima Yellin, Miriam Fried, Leeba Sullivan, Sara Morris, Giulio Kleiner, Daniel Stadlbauer, Jayeeta Dutta, Hui Xie, Manishkumar Patel, Kai Nie, Brian Monahan

Immunai Inc., New York, NY 10016, USA:
Adeeb Rahman

Oregon Health & Science University, Portland, OR 97239, USA:
William B. Messer, Catherine L. Hough, Sarah A.R. Siegel, Peter E. Sullivan, Zhengchun Lu, Amanda E. Brunton, Matthew Strand, Zoe L. Lyski, Felicity J. Coulter, Courtney Micheletti

Stanford University School of Medicine, Palo Alto, CA 94305, USA:
Holden Maecker, Bali Pulendran, Kari C. Nadeau, Yael Rosenberg-Hasson, Michael Leipold, Natalia Sigal, Angela Rogers, Andrea Fernandes, Monali Manohar, Evan Do, Iris Chang, Alexandra S. Lee, Catherine Blish, Henna Naz Din, Jonasel Roque, Linda N. Geng, Maja Artandi, Mark M. Davis, Neera Ahuja, Samuel S. Yang, Sharon Chinthrajah, Thomas Hagan, Tyson H. Holmes, Koji Abe

David Geffen School of Medicine at the University of California Los Angeles, Los Angeles CA 90095, USA:
Elaine F. Reed, Joanna Schaenman, Ramin Salehi-Rad, Adreanne M. Rivera, Harry C. Pickering, Subha Sen, David Elashoff, Dawn C. Ward, Jenny Brook, Estefania Ramires-Sanchez, Megan Llamas, Claudia Perdomo, Clara E. Magyar, Jennifer Fulcher

University of California San Francisco, San Francisco, CA 94115, USA:
David J. Erle, Carolyn S. Calfee, Carolyn M. Hendrickson, Kirsten N. Kangelaris, Viet Nguyen, Deanna Lee, Suzanna Chak, Rajani Ghale, Ana Gonzalez, Alejandra Jauregui, Carolyn Leroux, Luz Torres Altamirano, Ahmad Sadeed Rashid, Andrew Willmore, Prescott G. Woodruff, Matthew F. Krummel, Sidney Carrillo, Alyssa Ward, Charles R. Langelier, Ravi Patel, Michael Wilson, Ravi Dandekar, Bonny Alvarenga, Jayant Rajan, Walter Eckalbar, Andrew W. Schroeder, Gabriela K. Fragiadakis, Alexandra Tsitsiklis, Eran Mick, Yanedth Sanchez Guerrero, Christina Love, Lenka

Maliskova, Michael Adkisson, Aleksandra Leligdowicz, Alexander Beagle, Arjun Rao, Austin Sigman, Bushra Samad, Cindy Curiel, Cole Shaw, Gayelan Tietje-Ulrich, Jeff Milush, Jonathan Singer, Joshua J. Vasquez, Kevin Tang, Legna Betancourt, Lekshmi Santhosh, Logan Pierce, Maria Tecero Paz, Michael Matthay, Neeta Thakur, Nicklaus Rodriguez, Nicole Sutter, Norman Jones, Pratik Sinha, Priya Prasad, Raphael Lota, Saurabh Asthana, Sharvari Bhide, Tasha Lea, Yumiko Abe-Jones

Yale School of Medicine, New Haven, CT 06510, USA:
David A. Hafler, Ruth R. Montgomery, Albert C. Shaw, Steven H. Kleinstein, Jeremy P. Gygi, Dylan Duchen, Shrikant Pawar, Anna Konstorum, Ernie Chen, Chris Cotsapas, Xiaomei Wang, Charles Dela Cruz, Akiko Iwasaki, Subhasis Mohanty, Allison Nelson, Yujiao Zhao, Shelli Farhadian, Hiromitsu Asashima, Omkar Chaudhary, Andreas Coppi, John Fournier, M. Catherine Muenker, Khadir Raddassi, Michael Rainone, William Ruff, Syim Salahuddin, Wade L. Shulz, Pavithra Vijayakumar, Haowei Wang, Esio Wunder Jr., H. Patrick Young, Albert I. Ko, Gisela Gabernet

Yale School of Public Health, New Haven, CT 06510, USA:
Denise Esserman, Leying Guan, Anderson Brito, Jessica Rothman, Nathan D. Grubaugh, Kexin Wang, Leqi Xu

Baylor College of Medicine and the Center for Translational Research on Inflammatory Diseases, Houston, TX 77030, USA:
David B. Corry, Farrah Kheradmand, Li-Zhen Song, Ebony Nelson

Oklahoma University Health Sciences Center, Oklahoma City, OK 73104, USA:
Jordan P. Metcalf, Nelson I. Agudelo Higuita, Lauren A. Sinko, J. Leland Booth, Douglas A. Drevets, Brent R. Brown

University of Arizona, Tucson AZ 85721, USA:
Monica Kraft, Chris Bime, Jarrod Mosier, Heidi Erickson, Ron Schunk, Hiroki Kimura, Michelle Conway, Dave Francisco, Allyson Molzahn, Connie Cathleen Wilson, Ron Schunk, Trina Hughes, Bianca Sierra

University of Florida, Gainesville, FL 32611, USA:
Mark A. Atkinson, Scott C. Brakenridge, Ricardo F. Ungaro, Brittany Roth Manning, Lyle Moldawer

University of Florida, Jacksonville, FL 32218, USA:
Jordan Oberhaus, Faheem W. Guirgis

University of South Florida, Tampa FL 33620, USA:
Brittney Borresen, Matthew L. Anderson

The University of Texas at Austin, Austin, TX 78712, USA:
Lauren I. R. Ehrlich, Esther Melamed, Cole Maguire, Dennis Wylie, Justin F. Rousseau, Kerin C. Hurley, Janelle N. Geltman, Nadia Siles, Jacob E. Rogers, Pablo Guaman Tipan

IMPACC Network Competing Interests

The Icahn School of Medicine at Mount Sinai has filed patent applications related to SARS-CoV-2 serological assays, NDV-based SARS-CoV-2 vaccines, influenza virus vaccines, and therapeutics, listing Florian Krammer and Viviana Simon as co-inventors. Mount Sinai has spun out Kantaro to market SARS-CoV-2 serological tests and Castlevax to develop SARS-CoV-2 vaccines, with Florian Krammer as co-founder and scientific advisory board member of Castlevax. Florian Krammer has consulted for Merck, Curevac, Seqirus, GSK, Pfizer, 3rd Rock Ventures, Sanofi, Gritstone, and Avimex. His laboratory collaborates with Dynavax on influenza

vaccine development and with VIR on influenza virus therapeutics development. Ofer Levy is a named inventor on patents held by Boston Children's Hospital related to vaccine adjuvants and human in vitro platforms that model vaccine action. His laboratory has receives research support from GlaxoSmithKline (GSK) and he is a co-founder and advisor to *ARMR Sciences*, which develops preventative therapeutics against opioid overdose. Charles Cairns consults for bioMérieux and receives grant funding from the Bill & Melinda Gates Foundation. James A. Overton is a consultant at Knocean Inc. Jessica Lasky-Su is a scientific advisor for Precion Inc. Scott R. Hutton, Greg Michelloti, and Kari Wong are employees of Metabolon Inc. Vicki Seyfer-Margolis is employed by MyOwnMed. Nadine Rouphael reports grants or contracts with Merck, Sanofi, Pfizer, Vaccine Company, and Immorna. She has served on data safety monitoring boards for Moderna, Sanofi, Seqirus, Pfizer, EMMES, ICON, BARDA, and CyanVan Micron. She has received travel support from Sanofi and Moderna and honoraria from Virology Education and Krog Consulting. Chris Cotsapas is employed by Vesalius Therapeutics. Adeeb Rahman is employed by Immunai Inc. Steven Kleinstein consults for Peraton related to the ImmPort data repository. Nathan Grubaugh consults for Tempus Labs and the National Basketball Association. Akiko Iwasaki consults for 4BIO, Blue Willow Biologics, Revelar Biotherapeutics, RIGImmune, Xanadu Bio, and Paratus Sciences. Monika Kraft receives research funding from NIH, ALA, Sanofi, and AstraZeneca for asthma research. She consults for AstraZeneca, Sanofi, Chiesi, and GSK for severe asthma and is co-founder and CMO of RaeSedo, Inc., developing peptidomimetics for inflammatory lung disease. Esther Melamed receives research funding from Babson Diagnostics, honoraria from the Multiple Sclerosis Association of America, and has served on advisory boards for Genentech, Horizon, Teva, and Viela Bio. Carolyn Calfee receives research funding from NIH, FDA, DOD, Roche-Genentech, and Quantum Leap Healthcare Collaborative and consults for Janssen, Vasomune, Gen1e Life Sciences, NGMBio, and Cellenkos. Wade Schulz has collaborated with the Shenzhen Center for Health Information and the National Center for Cardiovascular Diseases in Beijing, is a technical consultant for Hugo Health, co-founder of Refactor Health, and has received grants from Merck and Regeneron Pharmaceuticals for COVID-19 research. Grace A. McComsey receives research grants from Redhill, Cognivue, Pfizer, and Genentech and consults for Gilead, Merck, and ViiV/GSK. Linda N. Geng receives research funding from Pfizer, Inc., through her institution. Catherine Hough receives research support from NIH and CDC. David Hafler has received research funding from Bristol-Myers Squibb, Novartis, Sanofi, and Genentech and consults for Bayer Pharmaceuticals, Repertoire Inc., Bristol Myers Squibb, Compass Therapeutics, EMD Serono, Genentech, Novartis Pharmaceuticals, and Sanofi Genzyme.

IMPACC network funding

NIH (3U01AI167892-03S2, 3U01AI167892-01S2, 5R01AI135803-03, 5U19AI118608-04, 5U19AI128910-04, 4U19AI090023-11, 4U19AI118610-06, R01AI145835-01A1S1, 5U19AI062629-17, 5U19AI057229-17, 5U19AI057229-18, 5U19AI125357-05, 5U19AI128913-03, 3U19AI077439-13, 5U54AI142766-03, 5R01AI104870-07, 3U19AI089992-09, 3U19AI128913-03, and 5T32DA018926-18); NIAID, NIH (3U19AI1289130, U19AI128913-04S1, and R01AI122220); NCATS, NIH UM1TR004528 and National Science Foundation (DMS2310836).

IMPACC network acknowledgments:

We thank the participants of the study for their voluntary enrollment and contribution of samples for this work. We acknowledge the assistance of the following individuals: Sanya Thomas, Mitchell Cooney, Shun Rao, Sofia Vignolo, and Elena Morrocchi (all from the CDCC); Arash Naeim, Marianne Bernardo, Sarahmay Sanchez, Shannon Intluxay, Clara Magyar, Jenny Brook, Estefania Ramires-Sanchez, Megan Llamas, Claudia Perdomo, Clara E. Magyar, and Jennifer A. Fulcher (all from the David Geffen School of Medicine at UCLA); members of the UCLA Center for Pathology Research Services and the Pathology Research Portal; M. Catherine Muenker, Dimitri Duvilaire, Maxine Kuang, William Ruff, Khadir Raddassi, Denise Shepherd, Haowei Wang, Omkar Chaudhary, Syim Salahuddin, John Fournier, Michael Rainone, and Maxine Kuang (all from the Yale School of Medicine). We thank the leadership of Boston Children's Hospital

including Drs. Wendy Chung, Gary Fleisher and Kevin Churchwell for their support for the Precision Vaccines Program. Dr. Augustine's and Becker's co-authorship of this report does not necessarily represent the official views of the National Institute of Allergy and Infectious Diseases, the National Institutes of Health or any other agency of the United States Government.

References

1. R. A. Spicer, R. Salek, C. Steinbeck, A decade after the metabolomics standards initiative it's time for a revision. Sci. Data 4, 170138 (2017).
2. L. W. Sumner, et al., Proposed minimum reporting standards for chemical analysis. Metabolomics 3, 211–221 (2007).
3. M. B. Members, et al., The Metabolomics Standards Initiative. Nat. Biotechnol. 25, 846–848 (2007).
4. J. Diray-Arce, et al., Multi-omic longitudinal study reveals immune correlates of clinical course among hospitalized COVID-19 patients. Cell Rep. Med. 4, 101079 (2023).
5. N. Rouphael, et al., Immunophenotyping assessment in a COVID-19 cohort (IMPACC): A prospective longitudinal study. Sci. Immunol. 6, eabf3733 (2021).
